# Optical Coherence Tomography Characterization of Coronary Lithotripsy Following Atherectomy for Treatment of Severely Calcified Lesions

**DOI:** 10.1161/CIRCINTERVENTIONS.126.016686

**Published:** 2026-07-08

**Authors:** Yohei Sotomi, Nehiro Kuriyama, Yutaka Tanaka, Seiji Yamazaki, Tomohiro Kawasaki, Kazushige Kadota, Takashi Muramatsu, Akihiko Takahashi, Takashi Ashikaga, Kenji Ando, Satoru Otsuji, Masaru Ishida, Shigeru Nakamura, Yoshiaki Ito, Raisuke Iijima, Gaku Nakazawa, Junya Shite, Junko Honye, Junya Ako, Hiroyoshi Yokoi, Ken Kozuma, Hiromasa Otake, Tomomi Yamada, Masato Nakamura

**Affiliations:** 1 Department of Cardiovascular Medicine, The University of Osaka Graduate School of Medicine, Japan (Y.S.).; 2 Department of Cardiology, Miyazaki Medical Association Hospital, Japan (N.K.).; 3 Department of Cardiology, Shonan Kamakura General Hospital, Japan (Y.T.).; 4 Department of Cardiology, Sapporo Higashi Tokushukai Hospital, Japan (S.Y.).; 5 Department of Cardiology, Tenjinkai Shin-Koga Hospital, Fukuoka, Japan (T.K.).; 6 Department of Cardiovascular Medicine and Department of Cardiology, Kurashiki Central Hospital, Okayama, Japan (K. Kadota).; 7 Department of Cardiology, Fujita Health University Hospital, Japan (T.M.).; 8 Department of Cardiology, Sakurakai Takahashi Hospital, Japan (A.T.).; 9 Department of Cardiology, Japanese Red Cross Musashino Hospital, Japan (T.A.).; 10 Department of Cardiology, Kokura Memorial Hospital, Kitakyushu, Japan (K.A.).; 11 Department of Cardiology, Higashi Takarazuka Satoh Hospital, Japan (S.O.).; 12 Division of Cardiology, Department of Internal Medicine, Iwate Medical University, Japan (M.I.).; 13 Department of Cardiology, Kyoto Katsura Hospital, Japan (S.N.).; 14 Department of Cardiology, Saiseikai Yokohamashi Tobu Hospital, Japan (Y.I.).; 15 Department of Cardiovascular Medicine (R.I.), Toho University Ohashi Medical Center, Japan.; 16 Division of Minimally Invasive Treatment in Cardiovascular Medicine (M.N.), Toho University Ohashi Medical Center, Japan.; 17 Department of Cardiology, Kindai University Hospital, Osaka, Japan (G.N.).; 18 Department of Cardiology, Osaka Saiseikai Nakatsu Hospital, Japan (J.S.).; 19 Department of Cardiology, Kikuna Memorial Hospital, Japan (J.H.).; 20 Department of Cardiovascular Medicine, Kitasato University Hospital, Japan (J.A.).; 21 Cardiovascular Center, Fukuoka Sanno Hospital, Japan (H.Y.).; 22 Department of Cardiology, Teikyo University Hospital, Japan (K. Kozuma).; 23 Division of Cardiovascular Medicine, Kobe University Graduate School of Medicine, Japan (H.O.).; 24 Department of Medical Innovation, The University of Osaka Hospital, Japan (T.Y.).

**Keywords:** atherectomy, calcium, humans, imaging, three-dimensional, lithotripsy, odds ratio, stents

## Abstract

**BACKGROUND:**

A combined strategy of atherectomy followed by intravascular lithotripsy (dual-prep strategy) for severely calcified lesions may optimize stent deployment, but mechanistic evidence from prospective optical coherence tomography (OCT) imaging is limited. This study was designed to comprehensively characterize patterns of calcium modification during a dual-preparation strategy and to explore imaging determinants of calcium fracture and stent expansion using serial and 3-dimensional OCT assessment.

**METHODS:**

This was a prespecified, multicenter, single-arm OCT substudy of the DUAL-PREP (atherectomy Devices and intravascUlAr Lithotripsy for the PREParation of heavy calcified coronary lesions) registry, enrolling patients with severely calcified lesions for whom combined treatment with atherectomy and intravascular lithotripsy was deemed preferable based on imaging findings. OCT was analyzed by an independent core laboratory. Multivariable models examined predictors of fracture and stenting performance, including calcium thickness, length, angle, calcified nodules, and morphology (circumferential, eccentric spiral, and eccentric straight types).

**RESULTS:**

OCT images were available for 114 of 118 patients (116 lesions; age, 75.9±9.1 years; 69.3% male). All lesions had an OCT calcium score of 4. Calcium fractures were uncommon after atherectomy (2/106 lesions [1.9%]) but markedly increased following intravascular lithotripsy (89/106 lesions [84.0%]; 2.05±1.59 fractures per lesion), with a further increase after stent implantation (101/109 lesions [92.7%]; 2.89±1.67 fractures per lesion). Final stent expansion index was 0.81±0.15 (eccentricity, 0.72±0.09; asymmetry, 0.39±0.15). Eccentric straight-type calcification was associated with a lower likelihood of fracture at final OCT (odds ratio, 0.07 [95% CI, 0.00–0.77]; *P*=0.029), whereas eccentric spiral and circumferential types showed higher fracture occurrence.

**CONCLUSIONS:**

This OCT analysis demonstrated a stepwise increase in calcium fracture during a dual-preparation strategy, with minimal fracture after atherectomy, a marked increase following intravascular lithotripsy, and a further increase after stent implantation. Calcium morphology may provide additional insight into fracture occurrence, supporting the potential value of 3-dimensional assessment in severely calcified lesions.

**REGISTRATION:**

URL: https://www.clinicaltrials.gov; Unique identifier: jRCT1032230384.

**What is Known**

- Severely calcified coronary lesions are associated with suboptimal stent expansion and worse clinical outcomes despite contemporary devices.
- A dual-preparation strategy using atherectomy followed by intravascular lithotripsy has demonstrated procedural safety, but the optical coherence tomography-based mechanisms of calcium fracture and determinants of effective stent expansion are not well defined.

**What the Study Adds**

- Dual preparation with atherectomy followed by intravascular lithotripsy resulted in favorable stent expansion with very low acute complication rates in severely calcified coronary lesions.
- Serial optical coherence tomography demonstrated a stepwise increase in calcium fracture during dual preparation, with minimal fracture after atherectomy, a marked increase after intravascular lithotripsy, and a further increase after stent implantation.
- Three-dimensional optical coherence tomography mapping showed that calcium morphology was associated with fracture occurrence and may provide additional insight beyond conventional cross-sectional calcium metrics.

**See Editorial by Ali et al**

The prevalence of severely calcified coronary lesions is increasing in parallel with an aging population and the rising incidence of diabetes and chronic kidney disease.^1,2^ Despite advances in drug-eluting stents, these lesions continue to pose major challenges, being associated with reduced procedural success and inferior long-term outcomes. Inadequate stent expansion—an established determinant of restenosis and stent thrombosis—remains particularly problematic; current consensus recommends a stent expansion index of >0.80 to optimize results.^3,4^ Accordingly, rigorous lesion preparation is considered indispensable. Available modalities include rotational and orbital atherectomy, intravascular lithotripsy (IVL), and modified balloons, each with inherent limitations. Atherectomy is constrained by guidewire bias, and larger burrs, while enhancing plaque debulking, heighten the risks of distal embolization, slow flow, and perforation.^5,6^ IVL, in contrast, enables wire-independent calcium modification but is limited by its bulky profile, restricted crossability in tortuous or long chronic total occlusions, and the disadvantage of absence of ablative capacity.

To overcome the limitations of individual devices, a combined strategy using atherectomy followed by IVL—referred to as the dual-preparation (dual-prep) approach—has been proposed to enhance safety and efficacy in complex lesion preparation. The DUAL-PREP (atherectomy Devices and intravascUlAr Lithotripsy for the PREParation of heavy calcified coronary lesions) registry demonstrated its short- and long-term clinical safety and effectiveness.^7,8^ Despite this angiographic and clinical success, intracoronary imaging may more sensitively detect suboptimal stent deployment and procedural complications. In addition, mechanistic insights into IVL-induced calcium fractures remain unexplored. Optical coherence tomography (OCT) reliably assesses coronary calcification. However, to date, most OCT studies have relied primarily on cross-sectional, slice-based calcium metrics (eg, arc, thickness, and related measures), whereas longitudinal continuity and 3-dimensional (3D) geometry of calcification across the lesion have been less systematically evaluated. This may overlook key mechanical contexts underlying lesion modification. This OCT substudy of the DUAL-PREP registry was designed to comprehensively characterize patterns of calcium modification during a dual-preparation strategy using atherectomy followed by IVL and to explore imaging determinants of calcium fracture and stent expansion using serial and 3D OCT assessment.

## Methods

### Data Availability

The data that support the findings of this study are available from the corresponding author upon reasonable request, following approval by the study sponsor/funder and in accordance with applicable data-sharing agreements.

### Study Design

The DUAL-PREP registry is a prospective, multicenter, single-arm study designed to assess the safety and efficacy of the dual-preparation strategy, atherectomy followed by IVL, before drug-eluting stent deployment in patients with severely calcified lesions.^7^

The inclusion criteria of the study were (1) age ≥18 years, (2) consent to participate in the study, (3) patients with severely calcified lesions for which treatment with IVL after other atherectomy devices may be preferable, based on imaging findings (OCT calcium score ≥3), and (4) calcification severity confirmed by angiography and OCT. The exclusion criteria were (1) patients who were participating in other clinical trials that may affect the results, and (2) patients ineligible for treatment with atherectomy or IVL.

The study was conducted in compliance with the ethical principles stated in the Declaration of Helsinki, and the study protocol was approved by central review by the institutional review board (Toho University Ohashi Hospital Ethics Board H23024, May 22, 2023). All patients provided written informed consent before participating in the study. This study was funded by Shockwave Medical, Inc. (Santa Clara, CA), which participated in discussions on the study design but was not involved in the data collection, study management, analysis, or interpretation of the data.

### Study Procedures

The designated atherectomy devices were the ROTAPRO Rotational Atherectomy System (Boston Scientific, Marlborough, MA) and the Diamondback 360 Coronary Orbital Atherectomy System (Abbott Vascular, Santa Clara, CA) for rotational and orbital atherectomy, respectively, while the Shockwave C2 Coronary IVL System (Shockwave Medical) was used for IVL. The treatment strategy has been described previously.^7^ In brief, the choice between rotational and orbital atherectomy was at the operator’s discretion, and all included lesions underwent atherectomy followed by IVL. The indication for IVL was limited to lesions with an OCT-based calcification score ≥3, in accordance with the catheter appropriate use criteria established by the Japanese Association of Cardiovascular Intervention and Therapeutics.^4^ Adjunctive balloon dilatation was performed at the operator’s discretion at multiple stages of the procedure. During the initial phases, including before baseline OCT and before IVL delivery, small-diameter balloon dilatation was primarily used to facilitate OCT catheter or IVL catheter delivery in severely calcified lesions, rather than to modify calcium. After IVL, further adjunctive balloon dilatation was permitted when additional lesion preparation was considered necessary. Finally, after stent implantation, balloon dilatation was performed at the operator’s discretion for stent optimization.

### Angiographic Evaluations

Quantitative coronary angiography was independently performed by the core laboratory (Micron, Inc, Osaka, Japan). Analyses were conducted at baseline, after atherectomy, after IVL, and at the final procedure. All analyses were performed offline using QAngio XA 7.3.102.0 (Medis Medical Imaging System, Leiden, the Netherlands). Further methodological details are provided in our previous publication.^7^

### OCT Acquisition and Analysis

OCT was recorded using the Dragonfly OpStar imaging catheter and OPTIS imaging system (Abbott Vascular) or the FastView imaging catheter and LUNAWAVE imaging system (Terumo Corporation, Tokyo, Japan). Imaging evaluation of coronary lesions with OCT was scheduled at 4 time points: baseline, postatherectomy, post-IVL, and final. OCT images were independently analyzed by the core laboratory (Micron, Inc) using the methods recommended in the expert consensus report for OCT.^9^ Analyses were performed offline using AptiVue ORW (Abbott Vascular) or LUNAWAVE Offline Viewer Software ver. 1.2 (Terumo Corporation, Tokyo, Japan). The distribution of calcified plaques, fractures, and dissections was illustrated using a spread-out vascular map view. Calcium morphology was classified into 3 types: circumferential, eccentric spiral, and eccentric straight. Further details are described in the Supplemental Methods.

### Statistical Analysis

All statistical analyses were performed using R software (version 4.3.2; R Foundation for Statistical Computing, Vienna, Austria). Categorical variables were expressed as counts (percentages). Continuous variables were expressed as mean (SD) or median (interquartile range). OCT data were analyzed per lesion. OCT data unavailable or unanalyzable at a given procedural stage due to suboptimal image quality were excluded from analyses for that stage. All analyses were conducted using complete-case data at each procedural stage; no imputation or assumptions regarding missing values were applied. Firth’s multivariable logistic regression models were used to explore factors associated with calcium fracture after IVL and stenting, stent underexpansion (stent expansion index <0.80), asymmetry (asymmetry index ≥0.30), and eccentricity (eccentricity index ≤0.70), by calculating multivariable-adjusted odds ratios (ORs) and 95% CIs. This method was chosen to reduce small-sample bias and to address data separation, thereby providing more reliable estimates in situations with limited events. Although there were only 2 patients with 2 lesions each, this mechanistic OCT analysis treated all lesions as independent, as the primary aim of the study was to gain mechanistic insights rather than to perform patient-level outcome analysis. Variables included in the model were selected by consensus among the research team, with attention to the risk of overfitting. These included calcium length (per mm), maximum calcium angle (per 10°), mean calcium angle (per 10°), maximum calcium thickness (per 0.1 mm), IVL balloon-to-artery ratio (IVL balloon/vessel diameter), stent-to-artery ratio (stent diameter/vessel diameter), number of calcium fractures after IVL, atherectomy device used (rotational atherectomy, orbital atherectomy, or both), type of calcified nodule (eruptive or noneruptive), calcium morphology (circumferential, eccentric spiral, or eccentric straight), and the presence of medial dissection after IVL. Statistical significance was defined as *P*<0.05.

## Results

### Study Subjects and Procedures

**Figure 1.**
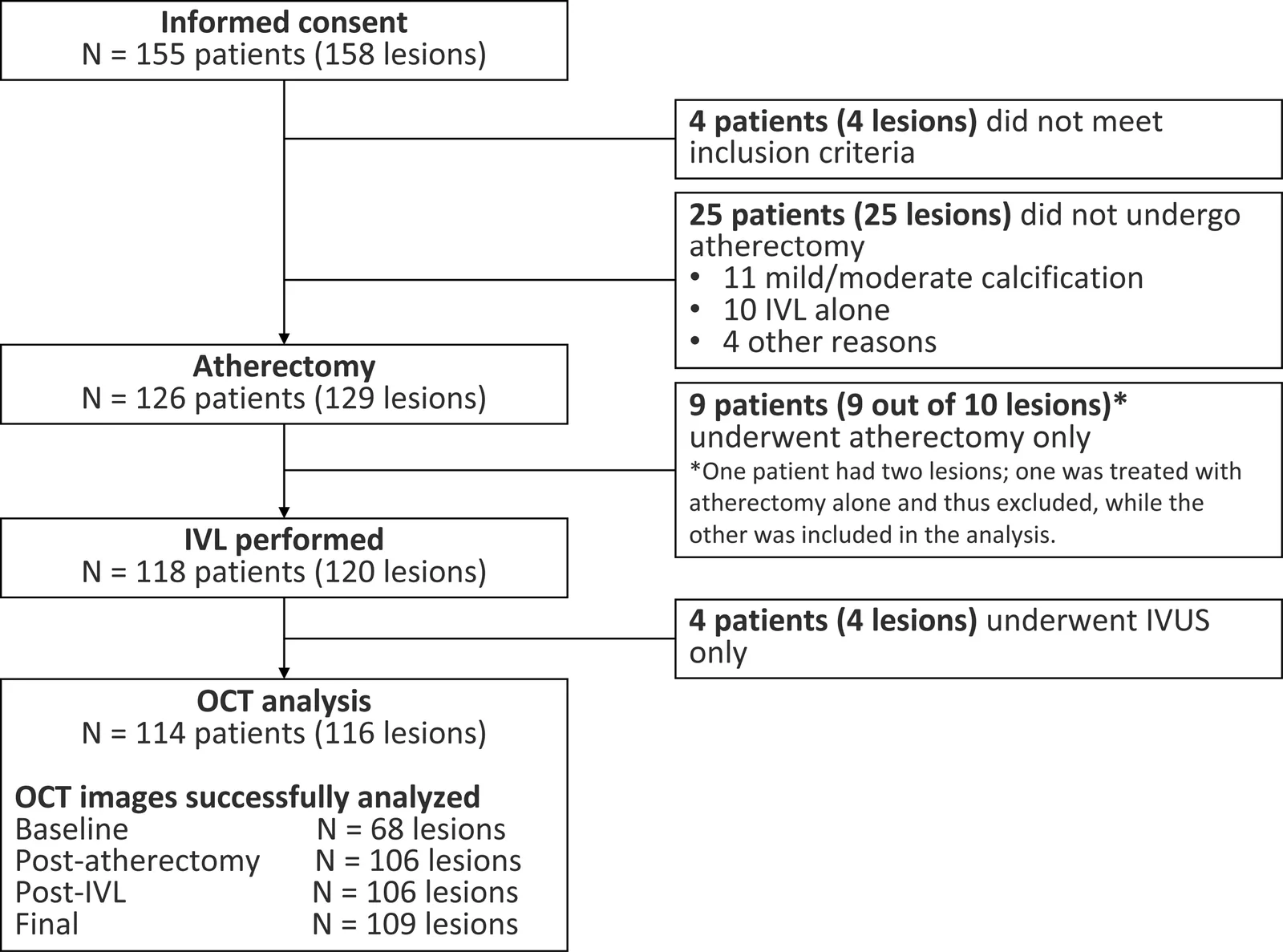
**Study flowchart.** Patients were enrolled from 20 sites in Japan between November 2023 and June 2024. IVL indicates intravascular lithotripsy; IVUS, intravascular ultrasound; and OCT, optical coherence tomography. * One patient had two lesions; one was treated with atherectomy alone and thus excluded, while the other was included in the analysis.

**Figure 2.**
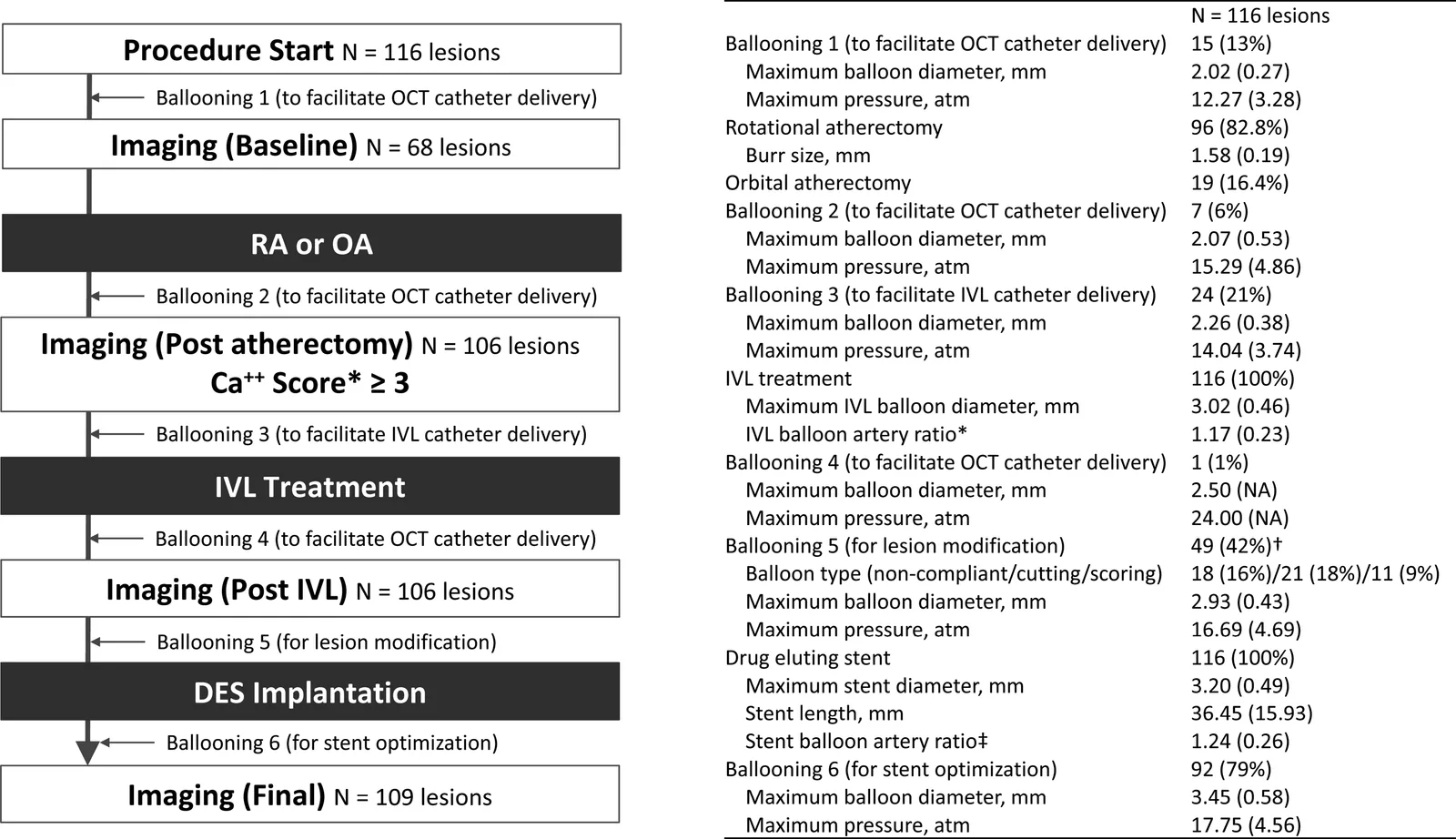
**Sequential procedural workflow and treatment details.** Procedural details are illustrated. Data are expressed as mean (SD) or number (percentage). *Intravascular lithotripsy (IVL) balloon-to-artery ratio (IVL balloon/mean reference vessel diameter by quantitative coronary angiography [QCA]). †Both a noncompliant balloon and a cutting balloon were used in 1 lesion. ‡Stent-to-artery ratio (stent diameter/mean reference vessel diameter by QCA). DES indicates drug-eluting stent; OA, orbital atherectomy; OCT, optical coherence tomography; and RA, rotational atherectomy.

A total of 118 patients with 120 lesions were enrolled at 20 participating centers between November 2023 and June 2024. A flowchart of the study is presented in Figure 1. OCT data were available for 114 patients (116 lesions). Patient demographics, lesion characteristics, and procedural characteristics are summarized in Tables S1 through S3 and Figure 2, respectively. Briefly, the mean age was 75.9±9.1 years; 69.3% of the patients were men, and 57.9% had diabetes. The radial approach was used in 74/114 (64.9%) patients. The target vessels included the left anterior descending artery in 75 (64.7%) lesions, the left circumflex artery in 3 (2.6%) lesions, the left main trunk in 6 (5.2%) lesions, and the right coronary artery in 36 (31.0%) lesions. All included lesions were classified as having severe calcification on angiography (Table S2). The sequential procedural workflow and treatment details are detailed in Figure 2. Rotational atherectomy was performed in 82.8% of lesions, while orbital atherectomy was used in 16.4%. IVL successfully crossed the target lesion in all patients. The mean IVL balloon diameter was 3.02±0.46 mm with an IVL balloon-to-artery ratio of 1.17±0.23. After IVL, adjunctive balloon dilatation was performed before post-IVL OCT imaging in 1 lesion (1%) and after post-IVL OCT imaging but before stent implantation in 49 (42%) lesions. Drug-eluting stents were implanted in all lesions, with a mean diameter of 3.20±0.49 mm and a mean length of 35.1±15.3 mm. Balloon dilatation after stenting was performed in 92 (79%) lesions. Slow flow occurred in only 1 case (0.8%) at the end of the procedure, and no perforation or other angiographic complications were observed.

### OCT Findings

#### Serial Observations and Stenting Performance

All lesions had an OCT calcium score of 4. Calcified nodules were present in 61 of 111 (55.0%) lesions (Table S2). Serial OCT analysis revealed a progressive increase in minimum lumen area from 1.67±0.63 mm^2^ at baseline to 5.71±2.05 mm^2^ at final (Table 1), with a concomitant reduction in area stenosis from 66.9±17.3% to 15.0±11.8%. Following atherectomy (pre-IVL), lumen dimensions increased modestly, while calcium burden metrics (maximum calcium thickness and arc) were largely unchanged. At final OCT, the minimum stent expansion index was 0.81±0.15, with a minimum stent area of 5.55±2.11 mm^2^, an eccentricity index of 0.72±0.09, and an asymmetry index of 0.39±0.15.

**Table 1.**
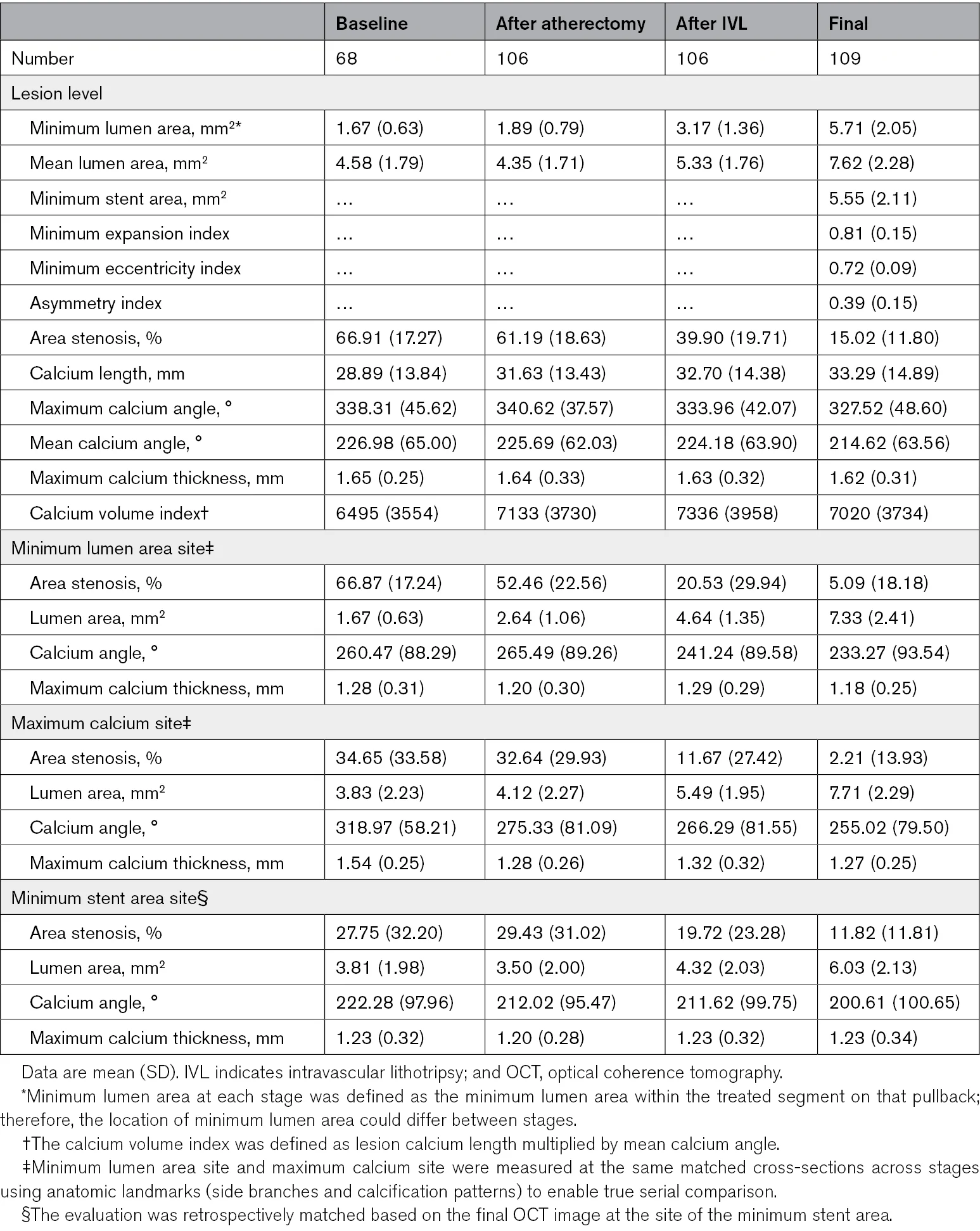
Serial Changes of OCT Characteristics

#### Fractures and Dissections

Serial OCT findings of calcium fractures and dissections are summarized in Table 2. Calcium fractures were observed after atherectomy in 2 lesions, which was uncommon at that stage, whereas IVL was associated with a marked increase in fracture detection (84.0% post-IVL), with a mean of 2.05±1.59 fractures per lesion. After stent deployment, fracture prevalence and burden showed a modest additional increase compared with post-IVL OCT (92.7% at final; 2.89±1.67 fractures per lesion), and fracture length and depth were also comparable between post-IVL and final assessments. This modest increase was accompanied by a high frequency of latent fractures, defined as fractures not visible after IVL but newly identified at the same sites at final, which were observed in 72.8% of evaluable lesions. Dissections were detectable as early as the postatherectomy stage. After IVL, the maximum and mean dissection angles were 25.9±15.1° and 13.8±6.8°, respectively. The dissection angle did not substantially increase from the postatherectomy to the post-IVL stage, suggesting that IVL predominantly contributed to calcium modification rather than expansion of the dissection extent.

**Table 2.**
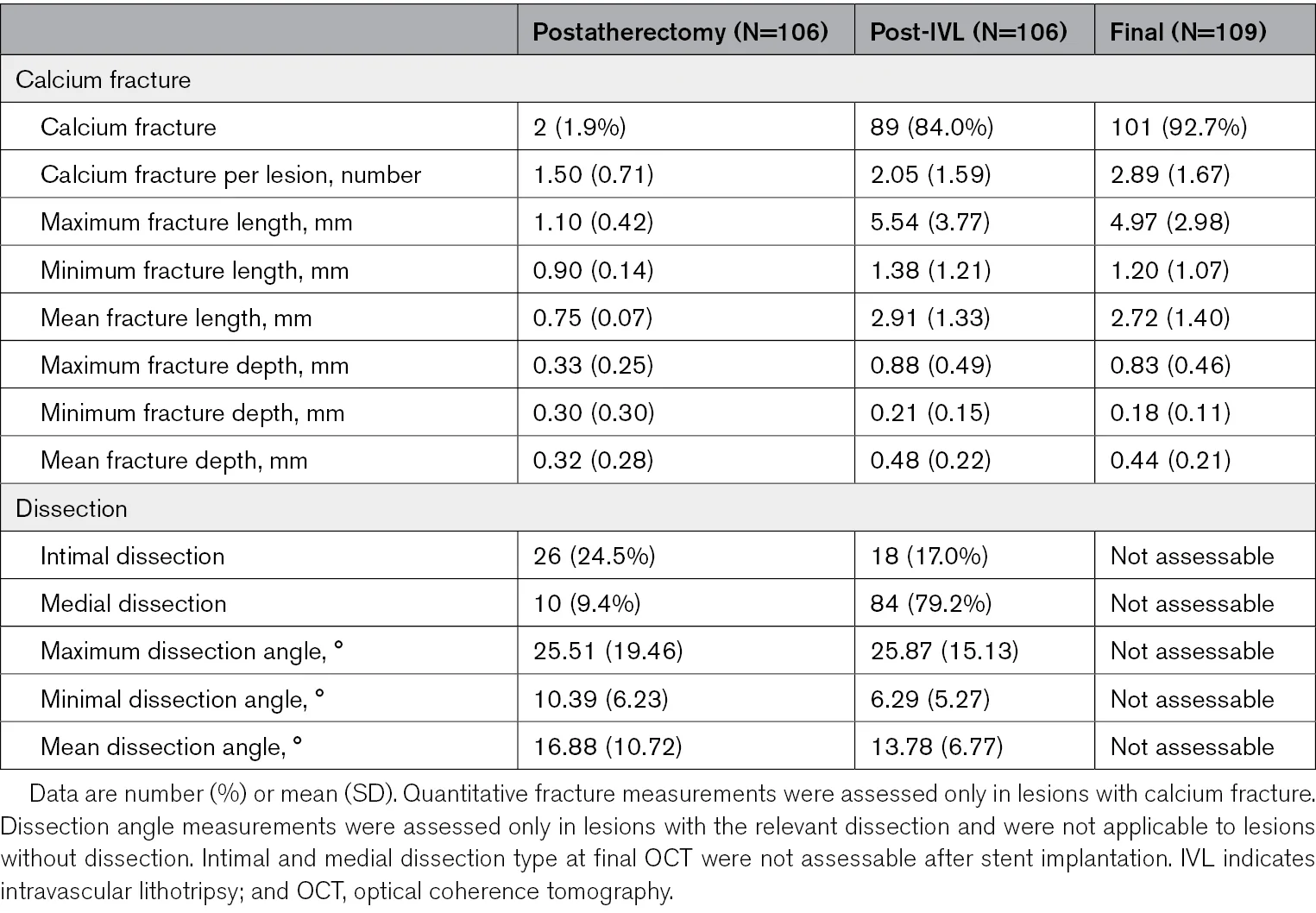
Serial Changes of Calcium Fractures and Dissections on OCT

#### Factors Associated With Fracture and Stenting Performance

Factors associated with calcium fracture and stenting performance were identified using Firth’s multivariable logistic regression models (Table 3 and Table S4). Among the evaluated OCT parameters, calcium morphology was associated with calcium fracture, whereas conventional cross-sectional metrics such as thickness or arc showed weaker associations in this cohort. Longer calcium length was significantly associated with stent asymmetry (OR, 1.05 [95% CI, 1.01–1.10]; *P*=0.024). Greater maximum calcium thickness was unexpectedly associated with a lower risk of stent underexpansion (per 0.1 mm, OR, 0.83 [95% CI, 0.71–0.96]; *P*=0.012). Figure S1 shows exploratory locally weighted scatterplot smoothing plots of minimum stent expansion index versus 4 lesion-level calcium metrics (maximum thickness, calcium length, maximum angle, and mean angle). Maximum calcium thickness showed a paradoxical positive association with stent expansion, whereas the other conventional metrics showed weaker and less consistent relationships. These visual findings were consistent with the multivariable analyses, in which calcium morphology was a more robust determinant of calcium fracture than slice-based calcium metrics. Atherectomy strategy was not independently associated with fracture occurrence or stenting performance after adjustment in the multivariable models, although unadjusted comparisons showed differences in some fracture metrics according to atherectomy strategy (Table S5).

**Table 3.**
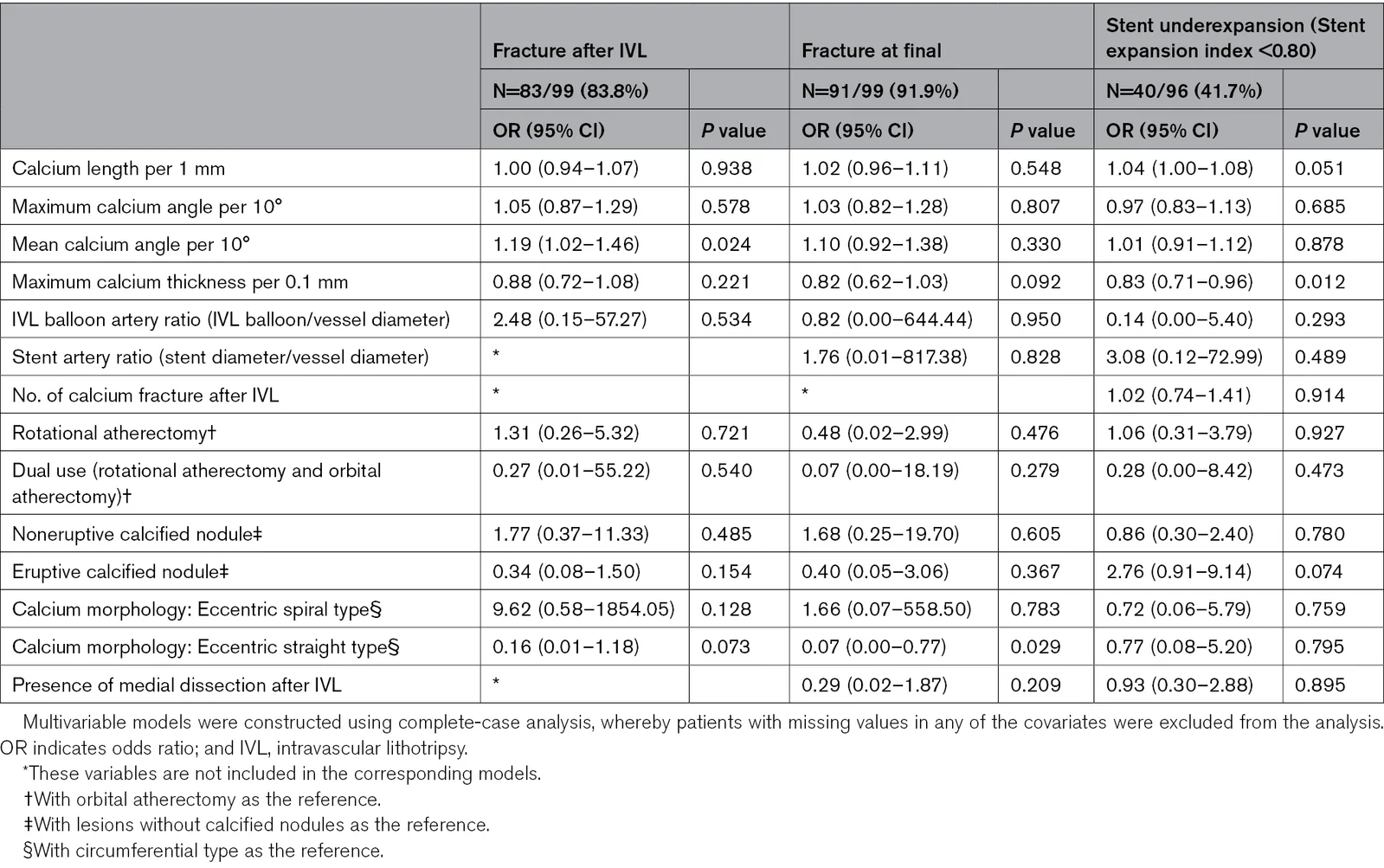
Firth’s Logistic Regression Analysis of Factors Associated With Calcium Fracture and Stenting Performance

**Figure 3.**
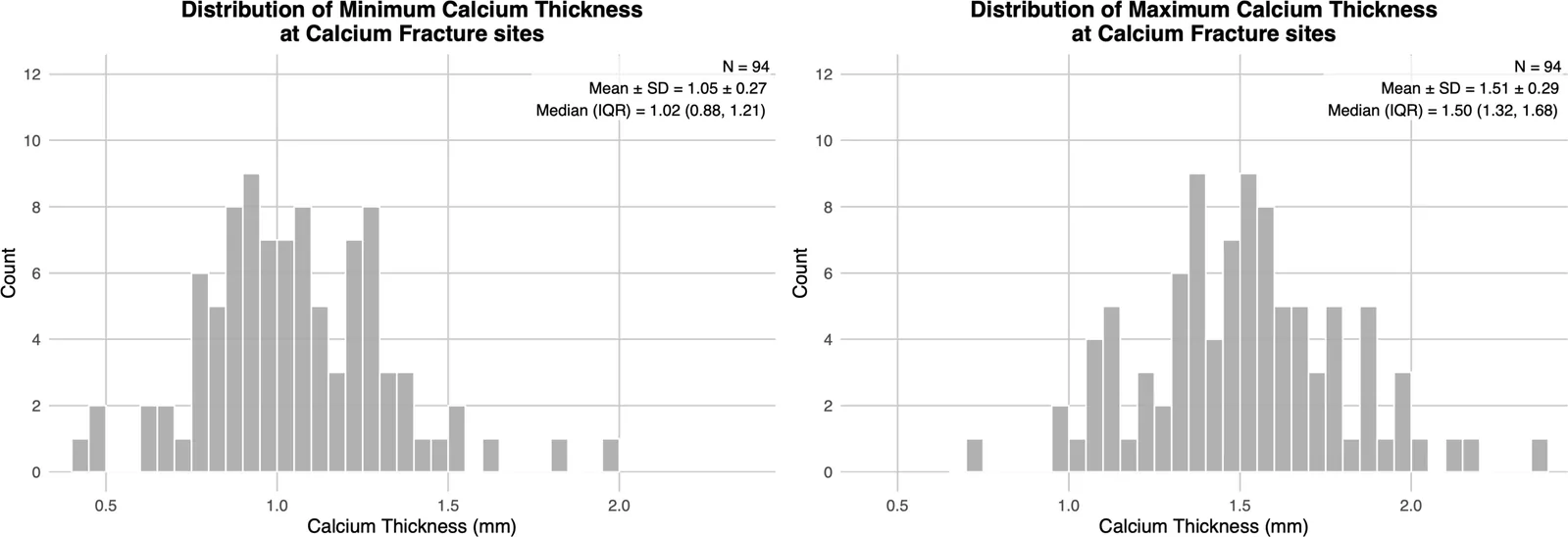
**Distribution of calcium thickness at calcium fracture sites.** This histogram shows the distribution of minimum (**left**) and maximum (**right**) calcium thickness measured at the sites where calcium fractures were identified on final optical coherence tomography. Thickness was defined as the total calcium thickness (ie, the full calcium depth from the luminal calcium surface to the abluminal calcium boundary) at the fracture site, and not as the depth of the fracture itself (fracture depth). In lesions with multiple fractures, calcium thickness was measured at each identifiable fracture site, and the minimum and maximum values among those measurements were summarized at the lesion level. Of the 101 lesions in which fractures were observed at Final, thickness measurements at the fracture site were feasible in 94 lesions; the remaining lesions were excluded because calcium thickness could not be reliably measured due to image limitations. IQR indicates interquartile range.

Figure 3 illustrates the distribution of minimum and maximum calcium thickness at sites where calcium fractures occurred, with a mean thickness of 1.05±0.27 mm and 1.51±0.29 mm, respectively.

**Figure 4.**
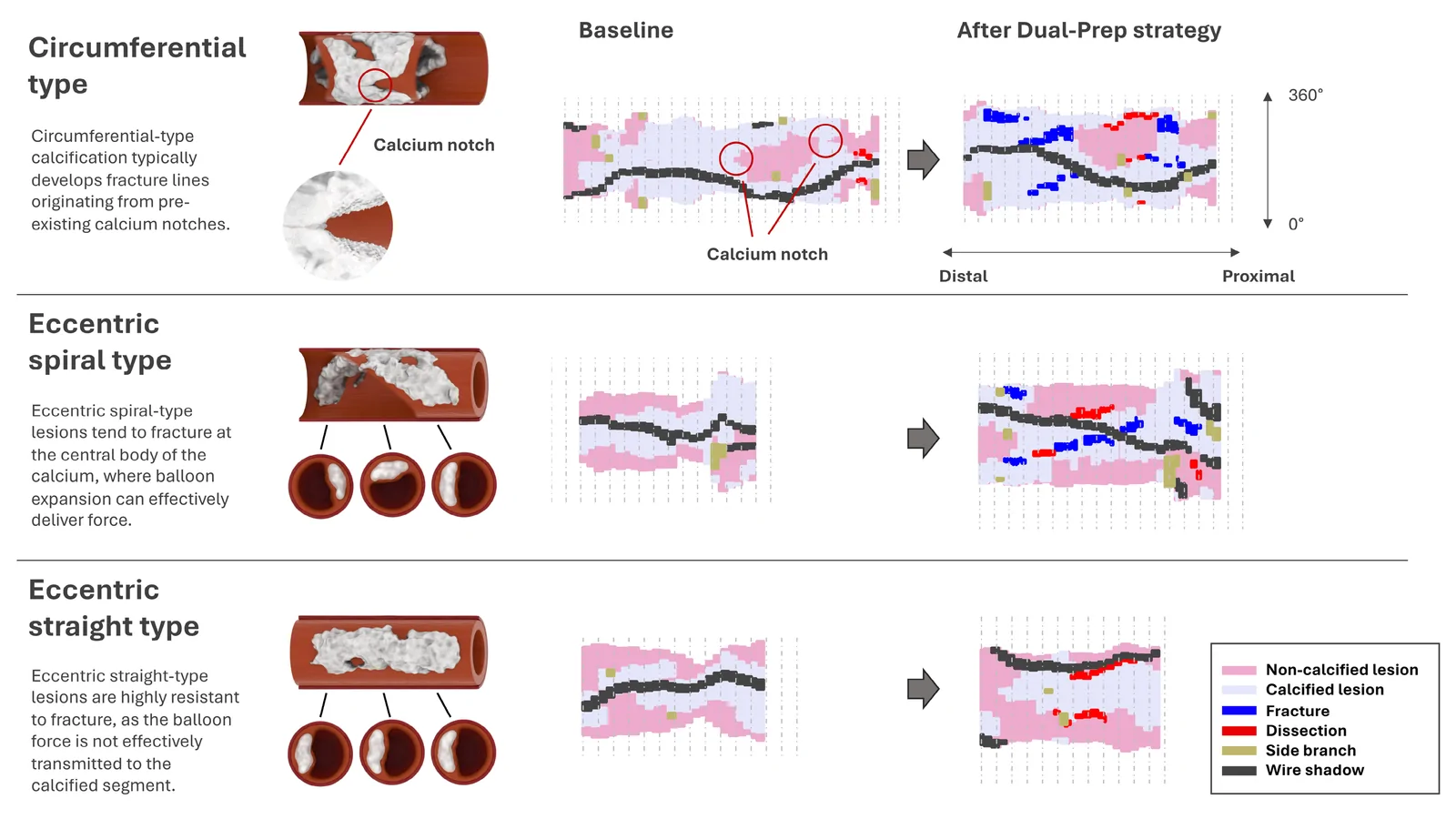
**Classification of calcified plaque morphology.** Calcified plaque morphology was, based on its shape and spatial orientation, classified into 3 patterns—circumferential, eccentric spiral, and eccentric straight types—each exhibiting distinct fracture behavior, as summarized within the figure. Calcium fractures (blue) frequently originated from preexisting calcium notches (red circle). Representative spread-out vascular maps are shown below to illustrate how each plaque type responds to the dual-preparation strategy. The horizontal axis represents the longitudinal vessel direction (from distal to proximal), and the vertical axis reflects circumferential plaque location, derived from optical coherence tomography angular data. The vertical extent at each position corresponds to the lumen area, allowing visual assessment of luminal changes along the vessel. Dissections were consistently observed along the longitudinal interface between calcified and noncalcified tissue, whereas fractures propagated from calcium notches following the spatial distribution of calcium. Dissections and fractures often occurred in continuity, likely reflecting regions where balloon force was concentrated during lesion preparation. Notably, eccentric straight-type lesions demonstrated dissections but rarely exhibited fractures, suggesting resistance to fracture propagation despite dual preparation. Spread-out vascular maps for all cases at each procedural step are provided in the Supplemental Material.

Visual assessment of spread-out vascular maps, generated for all OCT pullbacks, revealed 3 distinct patterns of calcification: circumferential, eccentric spiral, and eccentric straight (Figure 4; Figures S2 through S4). Circumferential-type calcification (98/112 [87.5%]) typically developed fracture lines originating from preexisting calcium notches, whereas eccentric spiral-type lesions (7/112 [6.3%]) tended to fracture at the central body of the calcium. In contrast, eccentric straight-type lesions (7/112 [6.3%]) were highly resistant to fracture. Fracture characteristics differed across subtypes (Table S6). The number of calcium fractures per lesion was highest in circumferential lesions and progressively lower in eccentric spiral and eccentric straight lesions (median [interquartile range], 3.00 [2.00–4.00] versus 2.00 [1.50–2.50] versus 1.00 [0.00–1.50], respectively; *P*=0.002). Maximum fracture depth also varied across subtypes, with deeper fractures in circumferential lesions compared with eccentric spiral and eccentric straight lesions (0.86±0.46 versus 0.55±0.28 versus 0.40±0.24 mm; *P*=0.038). In this cohort, dissections were consistently observed along the longitudinal interface between calcified and noncalcified tissue. Dissections and fractures often occurred in continuity, likely reflecting regions where balloon force was concentrated during lesion preparation.

Consistent with these visual observations, multivariable logistic regression analysis (Table 3) confirmed that, even after adjustment for other morphological and procedural factors, eccentric straight-type calcification tended to be associated with a lower likelihood of calcium fracture after IVL (OR, 0.16 [95% CI, 0.01–1.18]; *P*=0.073), and remained independently associated with a significantly lower likelihood at final (OR, 0.07 [95% CI, 0.00–0.77]; *P*=0.029). In contrast, the eccentric spiral-type achieved calcium fractures at rates comparable to circumferential lesions.

## Discussion

We report the following key findings. Under the dual-preparation strategy for severely calcified lesions: (1) this approach was feasible and resulted in favorable stent expansion; (2) calcium fracture increased stepwise across procedural stages, with minimal fracture after atherectomy, a marked increase following IVL, and a further increase after stent implantation; (3) calcium morphology was associated with fracture occurrence and may provide additional insight into fracture behavior; and (4) IVL was associated with frequent OCT-detected dissections, although the dissection angle remained limited.

To our knowledge, this is the first report to comprehensively characterize the lesion modification mechanisms of the dual-preparation strategy using OCT assessment. This study leveraged serial and 3D OCT imaging to systematically interrogate multiple aspects of calcium modification during dual preparation. Across these analyses, calcium morphology consistently outperformed traditional 2-dimensional metrics in explaining fracture behavior, supporting its important complementary role in lesion preparation.

### Therapeutic Dissection and Fracture

Across the Disrupt CAD series (N=675), IVL has shown poststenting calcium fracture rates identified by OCT imaging of 43% to 78.7%.^10,11^ In our analysis, fractures were observed in 84.0% of lesions after IVL and 92.7% at final, indicating that IVL combined with atherectomy in severely calcified lesions achieves very high fracture rates. The fracture rate on final OCT (92.7%) appears higher than that reported in many prior IVL series. Several cohort- and procedure-specific factors may contribute. This substudy focused on severely calcified lesions treated with a dual-preparation strategy, and the morphology distribution was enriched for circumferentially extensive calcification (circumferential type in 98/112 [87.5%] lesions), a configuration that fractured readily in our framework. In addition, the combined sequence (atherectomy followed by IVL) and subsequent optimization steps—including extensive ballooning and stent expansion—may facilitate not only fracture initiation by IVL but also propagation/opening of fracture planes along structural discontinuities, increasing the likelihood of demonstrable fracture on final OCT. Finally, systematic high-resolution OCT adjudication by an independent core laboratory using standardized criteria may increase fracture ascertainment compared with studies using different imaging protocols or definitions. Therefore, absolute fracture rates should be interpreted in the context of lesion severity, morphology distribution, and the cumulative procedural sequence, including adjunctive balloon dilatation and stent expansion, which may contribute to the propagation and visualization of calcium fractures beyond those induced by IVL alone.

Latent fractures, defined here as fractures not identifiable after IVL but newly apparent at the same sites on final OCT, were present in 72.8% of evaluable lesions. This OCT-based definition was conceived to mirror the histopathologic concept of microfracture. However, the finding should be interpreted with caution, as fractures not visible after IVL but apparent at final OCT may reflect increased detectability following subsequent mechanical expansion, rather than a distinct biological phenomenon.^12^ Although it is not strictly equivalent, it may provide a clinically relevant reference for elucidating mechanisms of calcium fracture associated with IVL.^10^ The absence of correlation between fracture number just after IVL and stenting performance may be explained by the high prevalence of latent fractures, which can inflate the total fracture count at final by adding fractures that become apparent only after stent optimization. In this context, fracture number is a coarse metric that may not capture the mechanistically relevant burden of calcium modification driving stent expansion.

**Figure 5.**
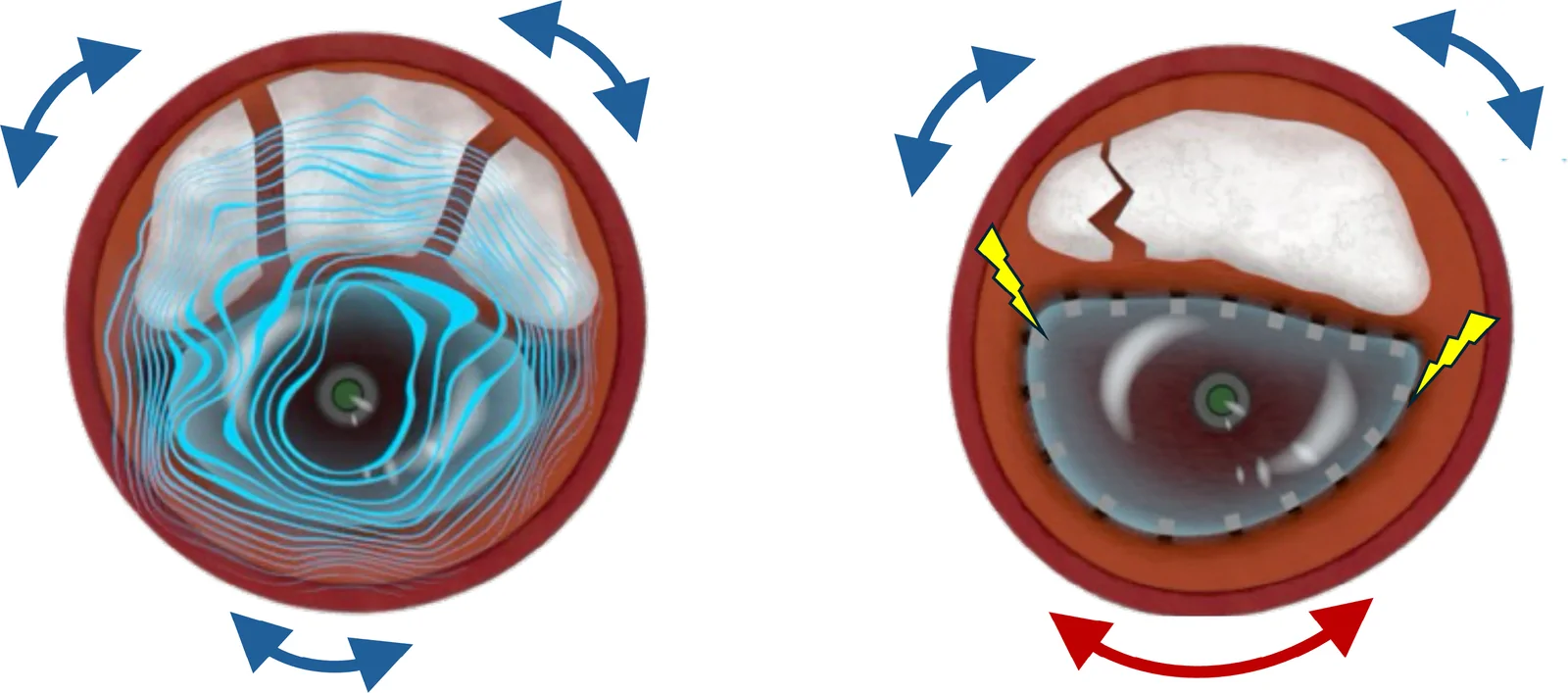
**Conceptual schematic of therapeutic dissection in severely calcified lesions.** This figure is a conceptual illustration (not a data-driven or computationally modeled stress map) summarizing a proposed mechanism by which intravascular lithotripsy (IVL)-based lesion modification may facilitate stent expansion. **Left**: With IVL at low balloon pressure, acoustic pulses initiate microdisruptions within calcium, and the resulting low-level circumferential (hoop) balloon tension may help gently open fracture planes and maintain apposition, yielding controlled fractures and limited dissections. **Right**: In contrast, aggressive high-pressure balloon overstretch may generate more focal peaks of circumferential stress, potentially leading to extensive dissections and excessive vessel stretch. Arrow size is illustrative and does not represent measured stress magnitude.

In the pooled Disrupt CAD analysis, type D to F dissections assessed by angiography occurred in 0.9% and perforations in 0.3% of cases.^10^ In contrast, our study reported no perforations and no severe dissections on angiography but identified medial dissections in 79.2% of lesions on OCT, with a limited maximum angle (25.9±15.1°).^7^ These OCT-detected dissections should not be regarded as equivalent to angiographic grade D to F dissections. The OCT-detected dissections, with a maximum angle of ≈25°, were limited in circumferential extent and would be expected to fall below the threshold of clinically significant angiographic dissections. If visible on angiography, these findings would most likely correspond to grades A to C rather than grades D to F. Because angiographic analyses generally focus on grades D to F, such minor dissections are not routinely included in reported dissection rates. This difference in detection and reporting thresholds likely explains the high frequency observed in our OCT-based assessment. Therefore, direct comparisons with angiographic studies reporting dissection rates of ≤1% may be misleading and should be avoided. IVL achieves calcium fracture at only 4 atm, minimizing injury to noncalcified segments and distributing vessel stretch more diffusely than high-pressure ballooning. This pattern may be considered a therapeutic dissection, in which controlled injury contributes to vessel expansion without adverse effects (Figure 5). In line with this concept, the extent of dissection (as assessed by dissection angle) was already evident after atherectomy and did not meaningfully increase after IVL on serial OCT. Importantly, these acute imaging observations were accompanied by reassuring longer-term clinical outcomes: 1-year clinical follow-up showed a major adverse cardiovascular event rate of 7.6% (cardiac death 2.5%, myocardial infarction 5.1%, and target vessel revascularization 5.1%), with 1 case of stent thrombosis.^8^ Although exploratory, these outcomes are encouraging and align with the notion that controlled, limited injury can facilitate expansion without an excess of adverse events.

Previous OCT studies have emphasized calcium thickness thresholds for fracture, ranging from 445 µm to 670 µm.^4,13–16^ However, in our heavily calcified cohort, calcium thickness was not clearly associated with fracture occurrence. Conversely, greater calcium thickness showed a paradoxical association with better stent expansion and a lower risk of stent underexpansion. One possible explanation is that circumferentially extensive calcification, often associated with greater thickness, facilitated multiple fractures, thereby enabling better stent expansion. These results may apply specifically to severely calcified lesions (OCT calcium score 4) and may not be generalizable to mildly or moderately calcified disease.

### Calcium Morphology

Calcium morphology emerged as an important determinant of fracture, representing a novel insight that complements rather than contradicts prior findings. A recent report, focusing mainly on thickness or arc, emphasized noncalcific factors—such as lipid composition or fibrous cap characteristics—as key predictors of expansion, suggesting that not the calcium itself but its surrounding structural context shapes vessel response.^17^ In that sense, our observation that the form and continuity of calcification critically determine fracture behavior can be viewed as the reverse side of the same phenomenon. Earlier studies relied primarily on cross-sectional analyses that treated each OCT slice independently, overlooking longitudinal vessel continuity.^13–16^ In contrast, we applied spread-out vascular mapping, enabling intuitive visualization of fracture distribution across the entire lesion and incorporation of 3D relationships between adjacent cross-sections. With this approach, we found that circumferential calcifications fractured more readily and that geometric discontinuities (notches) consistently served as initiation sites for fracture propagation—including native clefts such as side-branch ostia at the calcium–noncalcium interface. Mechanistically, balloon inflation generates circumferential (hoop) tensile stress, which concentrates at these discontinuities and facilitates crack initiation and propagation—analogous to a tear spreading easily from a notch.^18^ In eccentric spiral types, firm balloon apposition at the proximal and distal edges of the calcification helped transmit circumferential expansion into the plaque body, enabling effective fracture formation. Conversely, eccentric straight-type calcifications often resisted fracture even with IVL because circumferential (hoop) stress is less effectively transmitted into the main calcium body. Even in such lesions, acoustic pulses may still induce subtle microdisruptions that are not immediately apparent on OCT; however, without effective hoop-stress coupling, these microfractures may fail to widen or propagate into macroscopic fractures. In this context, we view acoustic pulse energy as the primary fracture-initiating mechanism, whereas the IVL balloon provides low-level circumferential (hoop) stress that can gently open preexisting fracture planes and maintain apposition, thereby facilitating fracture propagation and visualization. This morphology-centered, 3D analysis refines the understanding of how IVL interacts with calcified plaque and highlights the primacy of calcium architecture—and of stress concentration at structural notches—in determining procedural outcomes. Because lesion inclusion was restricted to cases with an OCT calcium score ≥3, variability in conventional parameters such as calcium arc, thickness, and length was limited, whereas morphology retained greater variability. Therefore, the present analysis should be interpreted as exploratory within a preselected high-calcium cohort.

Although comprehensive 3D OCT mapping is not yet routinely available in real time, the clinical value of the present findings is not limited to the availability of dedicated 3D software. The key message is that calcium continuity and geometry across the lesion—rather than single-slice 2-dimensional metrics alone—govern fracture behavior. This 3-dimension-informed concept can be translated into a pragmatic bedside interpretation: operators can approximate morphology by evaluating how the calcium arc evolves across consecutive frames—whether the eccentric calcium rotates along the vessel axis (eccentric spiral) or remains longitudinally aligned (eccentric straight), and whether circumferential segments and geometric discontinuities (notches) are present. In this way, understanding 3D morphology can refine the interpretation of conventional OCT imaging even when full 3D reconstruction is not performed.

### Clinical Implications and Future Direction

As a hypothesis-generating mechanistic study, these findings should be validated in independent cohorts; however, the internal consistency across multiple OCT analyses strengthens the biological plausibility of calcium morphology as a key determinant. At present, routine clinical workflows do not readily allow comprehensive 3D mapping from OCT in real time. Although computed tomography may offer advantages for 3D assessment, its spatial resolution and intraprocedural applicability remain limitations for capturing fine coronary calcium characteristics. Therefore, broader clinical translation of morphology-guided lesion preparation will likely require further advances in imaging and automated 3D reconstruction technologies.

### Strengths and Limitations

This study is the first to prospectively and comprehensively assess the mechanisms of the dual-preparation strategy—a combination of atherectomy followed by IVL—using systematic serial OCT imaging analyzed by an independent core laboratory. Notably, serial changes in calcified plaque characteristics were evaluated at 4 procedural time points, and the study included over 100 cases, representing, to our knowledge, the largest OCT-based imaging cohort to date in this specific dual-preparation setting. Importantly, while many prior OCT studies have primarily focused on cross-sectional, slice-based calcification metrics, our study additionally evaluated the continuity and spatial distribution of calcified plaques across serial cross-sections using spread-out vascular mapping, thereby providing novel mechanistic insights into calcium fracture.

Nevertheless, several limitations should be acknowledged. First, in this registry, the decision to use the dual-preparation strategy (atherectomy followed by IVL) was made by operators based on lesion complexity and imaging findings; therefore, selection bias cannot be excluded, and the cohort may not represent all-comers with calcified coronary disease. In addition, a portion of OCT images was unavailable or unanalyzable due to suboptimal quality, which may have also introduced selection bias. Second, this study lacked a comparator arm (eg, atherectomy alone, IVL alone, or alternative lesion-preparation strategies). Therefore, the present findings should be interpreted as mechanistic and descriptive within a dual-preparation cohort, and the incremental clinical value of this approach relative to standard-of-care strategies cannot be determined. Furthermore, because adjunctive balloon dilatation was performed pragmatically during lesion preparation, the vascular responses observed at each OCT checkpoint should be interpreted as the cumulative effect of the preparation sequence completed up to that stage rather than the isolated effect of a single device. In addition, atherectomy may contribute both to facilitation of subsequent device delivery and to lesion preparation within the overall sequential strategy, while the relative contribution of these mechanisms cannot be determined from the present study. Importantly, fractures immediately following atherectomy were minimal, highlighting its role in lesion preparation rather than as an independent fracture-generating intervention. Mechanistically, atherectomy modifies lesion geometry and reduces calcium thickness, facilitating subsequent IVL- or balloon-mediated fracture. These observations reinforce that the stepwise increase in fracture during dual preparation is primarily driven by IVL, with atherectomy serving a facilitative role. Third, our morphology assessment was reconstructed from standard OCT pullbacks using an offline spread-out mapping approach, and we acknowledge that comprehensive 3D mapping is not yet routinely feasible in real time in contemporary catheterization laboratory workflows. Therefore, immediate bedside implementation of morphology-guided lesion preparation would require further development and validation of automated reconstruction and coregistration technologies. Nevertheless, these findings provide important mechanistic and conceptual insights into calcium fracture that may refine interpretation of conventional 2-dimensional OCT and motivate future tool development. Fourth, superficial versus deep calcium was not formally evaluated. Although depth can be defined at the cross-sectional level, it varies along a lesion and there is no standardized lesion-level metric that aligns with our longitudinal 3D framework; dedicated studies with systematic depth mapping are warranted. Fifth, because calcium fractures were observed in the majority of lesions, the number of nonfracture cases was limited, which can lead to model instability and wide CIs when identifying predictors of fracture. We therefore used Firth’s penalized logistic regression; nevertheless, the multivariable models still seem potentially overparameterized relative to the number of lesions and the very small morphology subgroups. Furthermore, multiple exploratory imaging and regression analyses were conducted across various OCT-derived parameters and procedural stages, which may increase the risk of type I error. Therefore, nominally significant findings should be interpreted with caution. Sixth, given the multicenter design, procedural strategies and OCT findings may have been influenced by center-level or operator-level variability. Although formal adjustment for potential clustering effects across centers was not performed due to the limited number of lesions per center and the mechanistic, lesion-level focus of this study, future analyses could consider hierarchical modeling to account for such variability. Finally, it should be noted that the proposed calcium morphology classification was developed and evaluated within the same data set. Future studies are warranted to perform external validation and reproducibility assessment in independent cohorts to strengthen confidence in its robustness, generalizability, and potential clinical utility.

### Conclusions

This OCT analysis revealed high rates of calcium fracture and limited medial dissection without vessel perforation, resulting in favorable stent expansion. Calcium fracture increased stepwise during the dual-preparation strategy, with minimal fracture after atherectomy, a marked increase following IVL, and a further increase after stent implantation. Calcium morphology may provide additional insight into fracture occurrence, supporting the potential value of 3D assessment by emphasizing longitudinal calcium continuity and geometry across consecutive frames.

## Article Information

### Acknowledgments

The authors thank Yoshito Nakamoto, Kou Tachikawa, Keiji Noutomi, and Naoki Matsukawa (Micron, Inc, Osaka, Japan) for the optical coherence tomography (OCT) core laboratory analysis, and Asahi Funayama and Kanako Imamura (Micron, Inc, Tokyo, Japan) for the study management. English editing was assisted by the open artificial intelligence (AI) chatbot ChatGPT (OpenAI, San Francisco, CA), which was used solely for language editing and did not influence the data analysis or interpretation.

### Disclosures

Dr Sotomi has received grants from Abbott Medical Japan, Abiomed, Biosense Webster, Boston Scientific Japan, Johnson & Johnson, Kaneka, Nipro, and Terumo, and honoraria from Abbott Medical Japan, Boston Scientific Japan, Kaneka Medics, Nipro, Terumo, and Shockwave Medical Japan. Dr Tanaka has received honoraria from Shockwave Medical Japan. Dr Kawasaki has received honoraria from Abbott Medical Japan, Boston Scientific Japan, and Terumo. Dr Muramatsu has received honoraria from Abbott Medical Japan, Boston Scientific Japan, Shockwave Medical Japan, and Terumo. Dr Ando has received honoraria from Abbott Medical Japan, Medtronic Japan, BIOTRONIK JAPAN, Japan Lifeline, and Novartis Pharma. Dr Otsuji has received honoraria from Abbott Medical Japan, Boston Scientific Japan, and Shockwave Medical Japan. Dr Ishida has received honoraria from Abbott Medical Japan, Boston Scientific Japan, and Terumo. Dr Ito has received honoraria from Boston Scientific Japan, Abbott Medical Japan, and Terumo. Dr Iijima has received honoraria from Boston Scientific Japan and Terumo. Dr Nakazawa has received honoraria from Abbott Medical Japan, Boston Scientific Japan, Terumo, and Shockwave Medical Japan. Dr Ako has received honoraria from Shockwave Medical Japan, Abbott Medical Japan, and Nipro. Dr Kozuma has received honoraria from Shockwave Medical Japan, Medtronic, Boston Scientific Japan, and Abbott Medical Japan, and serves as a board member of *Cardiovascular Intervention and Therapeutics*. Dr Otake has received grants from Abbott Medical Japan and honoraria from Abbott Medical Japan and Terumo. Dr Nakamura has received consulting fees from Shockwave Medical Japan, honoraria from Boston Scientific Japan, Terumo, and Shockwave Medical Japan, and endowments from Boston Scientific Japan and Terumo. The other authors report no conflicts.

### Supplemental Material

Supplemental Methods

Figures S1–S4

Tables S1–S6

References 19–22

## Supplementary Material
