# Supplementary material for: Optical Coherence Tomography Characterization of Coronary Lithotripsy Following Atherectomy for Treatment of Severely Calcified Lesions
Source: Circ Cardiovasc Interv. 2026 Jul 8;19(9):e016686. doi: 10.1161/CIRCINTERVENTIONS.126.016686 (PMC13574353; doi:10.1161/CIRCINTERVENTIONS.126.016686)
Supplement: Supplementary file 1 [file hcv-19-e016686-s001.pdf]

SUPPLEMENTAL MATERIAL

Optical coherence tomography characterization of coronary lithotripsy following atherectomy for treatment of severely calcified lesions

Supplemental Methods

OCT acquisition and analysis

OCT was recorded using the Dragonfly™ OpStar imaging catheter and OPTIS imaging system (Abbott Vascular, Santa Clara, California) or the FastView® imaging catheter and LUNAWAVE® imaging system (Terumo Corporation, Tokyo, Japan). Imaging evaluation of coronary lesions with OCT was scheduled at four time points: baseline, post-atherectomy, post-IVL, and Final. Final OCT was defined as the OCT pullback acquired after stent implantation and completion of all planned stent optimization, including post-dilatation and/or proximal optimization technique when performed. During image acquisition, undiluted iodinated contrast or low-molecular-weight dextran was injected to achieve blood clearance, and an automated pullback system was used.

A broad set of OCT-derived parameters reflecting calcium burden, spatial distribution, and 3D morphology were prespecified for analysis, including calcium thickness, arc, length, presence of nodules, fracture characteristics, and calcium morphology derived from spread-out vascular mapping. These parameters were evaluated to identify imaging features associated with calcium fracture and stent expansion.

OCT images were independently analyzed by the core lab (Micron, Inc., Osaka, Japan) using the methods recommended in the expert consensus report for OCT. [9] Analyses were performed offline using AptiVue™ ORW (Abbott Vascular, Santa Clara, California) or LUNAWAVE® Offline Viewer Software ver. 1.2 (Terumo Corporation, Tokyo, Japan). Quantitative OCT assessment was performed at 1-mm intervals, while qualitative assessment of calcium fracture and dissection was performed frame by frame.

The stented segments, along with the 5-mm segments adjacent to both proximal and distal edges (reference segments), and their corresponding lesions at baseline, after atherectomy, and after IVL were analyzed. The reference lumen area was estimated as the mean lumen area of the reference segments, or, if a 5-mm segment was not available, the most proximal and most distal cross-sectional areas of the analyzed segment. Area stenosis (%) was calculated as (1 − [minimal lumen area / projected reference lumen area by linear model]) × 100. [19] The minimum stent expansion index was defined as the minimum stent area divided by the projected reference lumen area by linear model. [19] The asymmetry index was defined as (1 − [minimal lumen diameter / maximal lumen diameter]) across the stented segment, with lesions considered asymmetrical when the index was ≥0.3. [20] The eccentricity index, a measure of cross-sectional circularity, was calculated as the minimal lumen diameter divided by the maximal lumen diameter from the same cross-section. [20] Minimum eccentricity index was defined as the lowest value within the entire lesion, with a value ≤0.7 considered eccentric.

Calcified plaque was defined as a signal-poor or heterogeneous region with borders that were either clearly delineated or obscured by signal attenuation. Each calcific plaque within the target lesion was assessed for calcium angle (measured from the lumen's center of mass), maximum calcium

thickness, and longitudinal calcium length. Calcium edges were identified using full-frame analysis rather than 1-mm interval assessment. The calcium volume index was calculated as mean calcium angle (degrees) × calcium length (millimeters). When mixed calcified plaque caused signal attenuation and obscured plaque boundaries, calcium edges were interpolated from adjacent cross-sections and used for measurement.

An OCT-based calcium scoring system—assigning 2 points for a calcium angle >180°, 1 point for plaque length >5 mm, and 1 point for calcium thickness >0.5 mm—was systematically applied.[4] Calcium fracture was defined as a new disruption or discontinuity in the calcium sheet identified on OCT after atherectomy, IVL or stenting. "Latent fracture" was defined as fractures not identifiable after IVL but newly apparent at the same sites on Final OCT. To assess the number of fractures per lesion, fracture lines were tracked for continuity on a frame-by-frame basis throughout the lesion. For fracture-site thickness analysis, calcium thickness was measured directly at sites where calcium fracture was identified and was defined as the total calcium thickness from the luminal calcium surface to the abluminal calcium boundary, rather than as the depth of the fracture itself. Fracture depth was defined separately as the distance from the luminal calcium surface to the deepest point of the fracture. Dissection was categorized as intimal or medial, and the dissection angle was measured from the lumen's center of mass. Calcium fracture and dissection were adjudicated on OCT after atherectomy, after IVL and at Final OCT. The distribution of calcified plaques, fractures, and dissections was illustrated using a spread-out vascular map view. Calcified nodules were categorized as eruptive or non-eruptive.[21]

Morphology classification was developed through an iterative visual review of the spread-out vascular maps generated from all OCT pullbacks, focusing on longitudinal calcium continuity and circumferential distribution across consecutive frames. Based on recurring and readily recognizable configurations, calcium morphology was classified into three patterns: circumferential, eccentric spiral, and eccentric straight. To enable reproducible categorization, lesions were first classified as circumferential versus eccentric using a rule-based algorithm based on calcium arc measurements throughout the lesion: lesions with a maximum calcium arc ≥270° and/or a mean calcium arc ≥180° were classified as circumferential, whereas those with a maximum arc <270° and a mean arc <180° were classified as eccentric. Among eccentric lesions, spiral and straight types were further subclassified by visual assessment of calcium distribution on the spread-out vascular map—lesions with calcium distributed in a helical pattern were classified as spiral type, whereas those with calcium aligned in a linear, horizontal direction were classified as straight type.

Reproducibility assessment for OCT fracture and calcium morphology

Inter- and intra-observer variability were assessed for both OCT-based fracture assessment and calcium morphology classification. For interobserver agreement, two independent observers, blinded to clinical and procedural information, performed the assessments independently. For intra-observer agreement, one observer repeated the assessments after a 4-week washout period, blinded to the initial assessments.

For OCT-based fracture assessment, reproducibility was evaluated for both qualitative and quantitative parameters. For qualitative assessment across 306 calcified cross-sections, agreement for the presence or absence of fracture was assessed using Cohen's kappa statistics, and agreement

for the number of fractures per cross-section was assessed using quadratic weighted kappa statistics. Interobserver agreement for fracture presence/absence was moderate ($\kappa = 0.616$), whereas intra-observer agreement was substantial ($\kappa = 0.862$). For fracture number per cross-section, interobserver agreement was moderate (quadratic weighted $\kappa = 0.602$), and intra-observer agreement was substantial (quadratic weighted $\kappa = 0.819$). For quantitative assessment, reproducibility of fracture length and fracture depth was evaluated using intraclass correlation coefficients. Fracture length was measured in 10 runs, yielding an interobserver ICC of 0.661 and an intra-observer ICC of 0.994. Fracture depth was assessed in 35 cross-sections for interobserver comparison and 40 cross-sections for intra-observer comparison, with corresponding ICCs of 0.824 and 0.986, respectively. Overall, OCT-based fracture assessment showed acceptable reproducibility, with particularly high agreement for repeated measurements by the same observer.

For morphology classification, because circumferential morphology was determined deterministically by the prespecified arc-based rule, agreement analyses focused on the visual subclassification of eccentric lesions (spiral vs straight) based on the spread-out vascular map. Cohen's kappa statistics were calculated for the binary classification, yielding an interobserver κ of 0.714 and an intra-observer κ of 0.857, indicating substantial agreement.

Supplemental Figures and Figure Legends

Figure S1. LOESS plots of minimum stent expansion index versus lesion-level calcium metrics

Scatter plots showing the relationship between minimum stent expansion index (y-axis) and four lesion-level calcium metrics (x-axis): maximum calcium thickness (upper left), calcium length (upper right), maximum calcium angle (lower left), and mean calcium angle (lower right). A locally weighted scatterplot smoothing (LOESS) curve (blue line) with 95% confidence interval (gray shaded area) is overlaid in each panel to illustrate the observed trends. The horizontal dashed line indicates a minimum stent expansion index of 0.80. These plots are presented as exploratory visualizations of unadjusted associations. The limited sample size as well as sparse data distribution at the extremes may affect the stability of the observed patterns. The non-linear associations should be interpreted cautiously, and the plots are intended to provide descriptive, hypothesis-generating insights rather than definitive evidence.

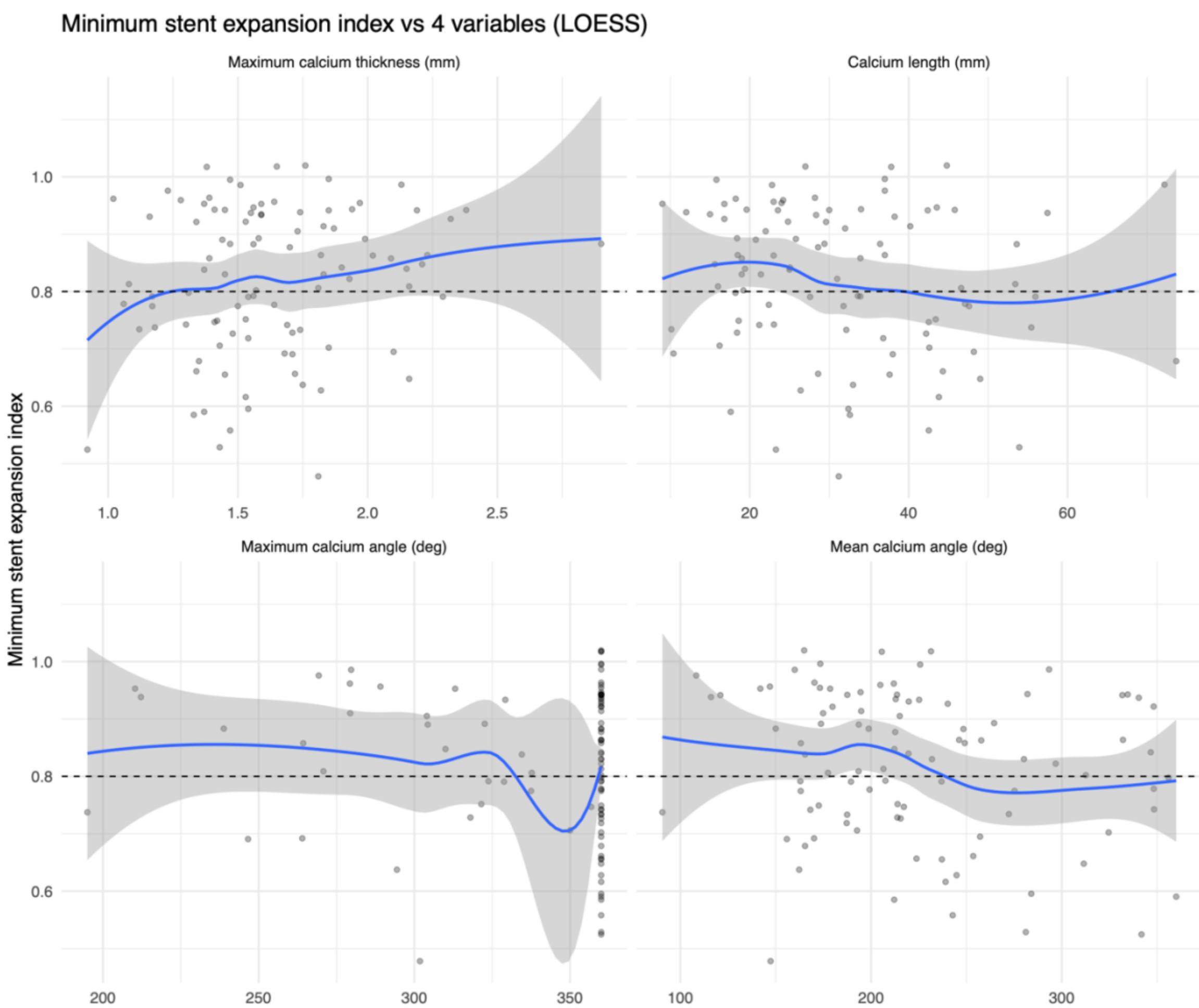

Figure S2. Spread-out vascular maps for circumferential type calcification

Spread-out vascular maps illustrate the circumferential and longitudinal distribution of calcified plaque, calcium fractures, and dissections throughout the dual preparation strategy (atherectomy followed by intravascular lithotripsy [IVL]) and subsequent stent deployment. Panels A–E present a continuous display of the circumferential-type cohort (n = 98) for readability, with lesions ordered by calcium volume index in descending order (highest to lowest). Within each panel, images are presented chronologically from left to right: baseline, post-atherectomy, post-IVL, and final. The horizontal axis represents the vessel's longitudinal direction (distal to proximal), while the vertical axis indicates circumferential plaque location reconstructed from OCT-derived angular data. The vertical extent at each longitudinal position corresponds to the lumen area, enabling visual assessment of luminal changes along the vessel. Circumferential-type calcification typically develops fracture lines originating from pre-existing calcium notches.

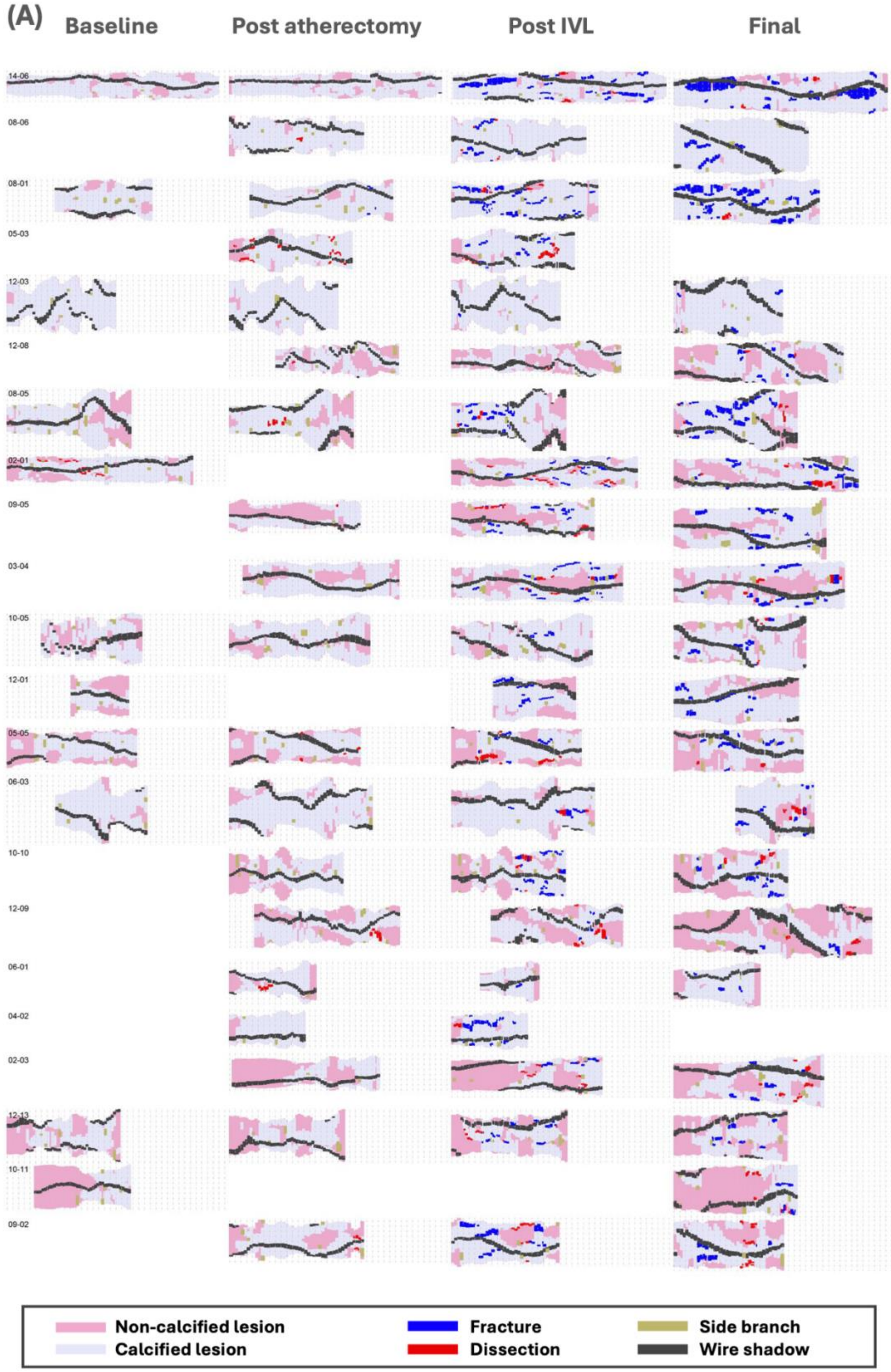
(A)
Baseline
Post atherectomy
Post IVL
Final
14-06
08-06
08-01
05-03
12-03
12-08
08-05
02-01
09-05
03-04
10-05
12-01
05-05
06-03
10-10
12-09
06-01
04-02
02-03
12-13
10-11
09-02
Non-calcified lesion
Calcified lesion
Fracture
Dissection
Side branch
Wire shadow

(B)

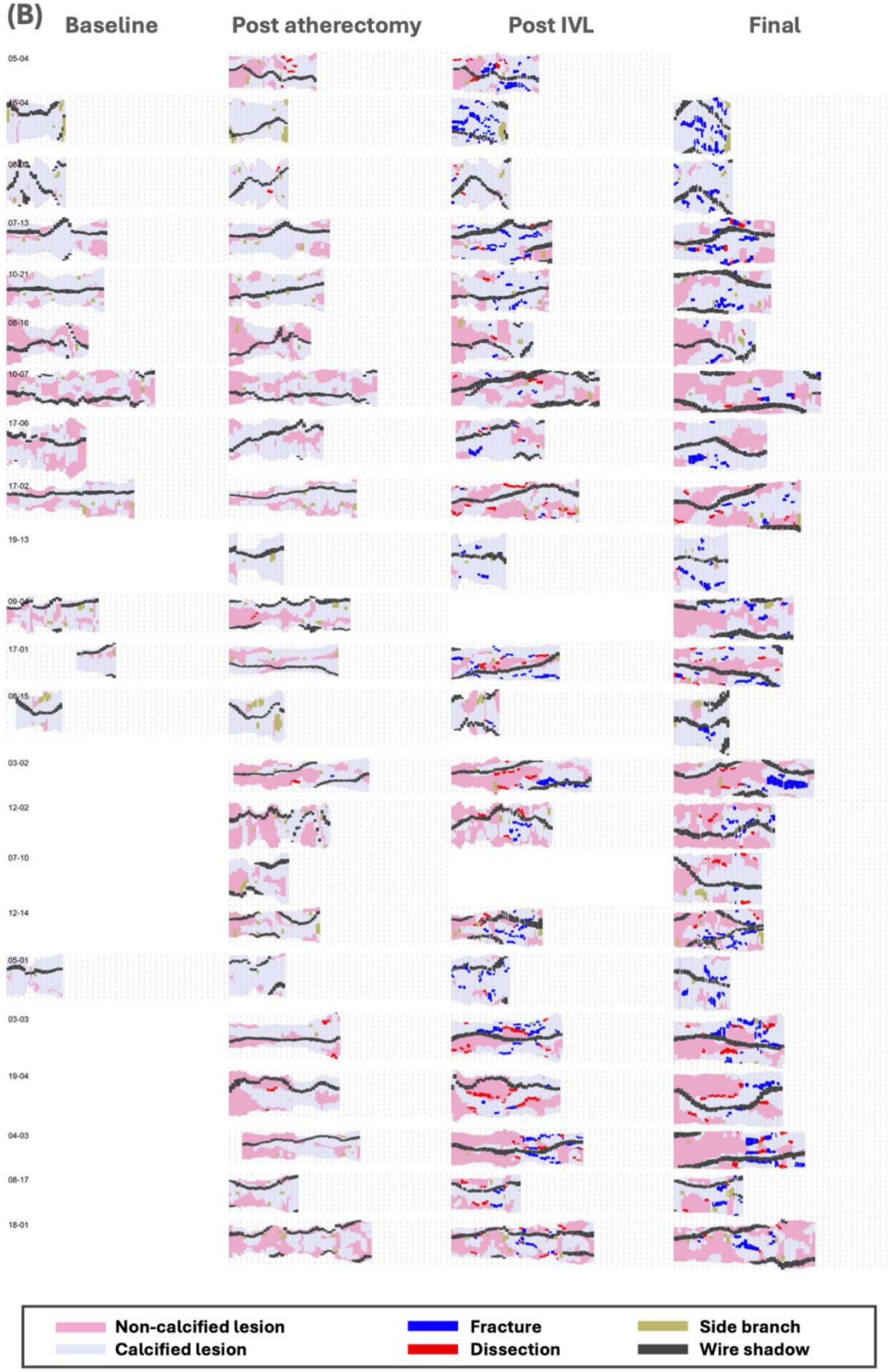

Baseline
Post atherectomy
Post IVL
Final
05-04
07-13
10-21
08-16
10-07
17-06
17-02
19-13
17-01
03-02
12-02
07-10
12-14
05-01
03-03
19-04
04-03
08-17
18-01
Non-calcified lesion
Calcified lesion
Fracture
Dissection
Side branch
Wire shadow

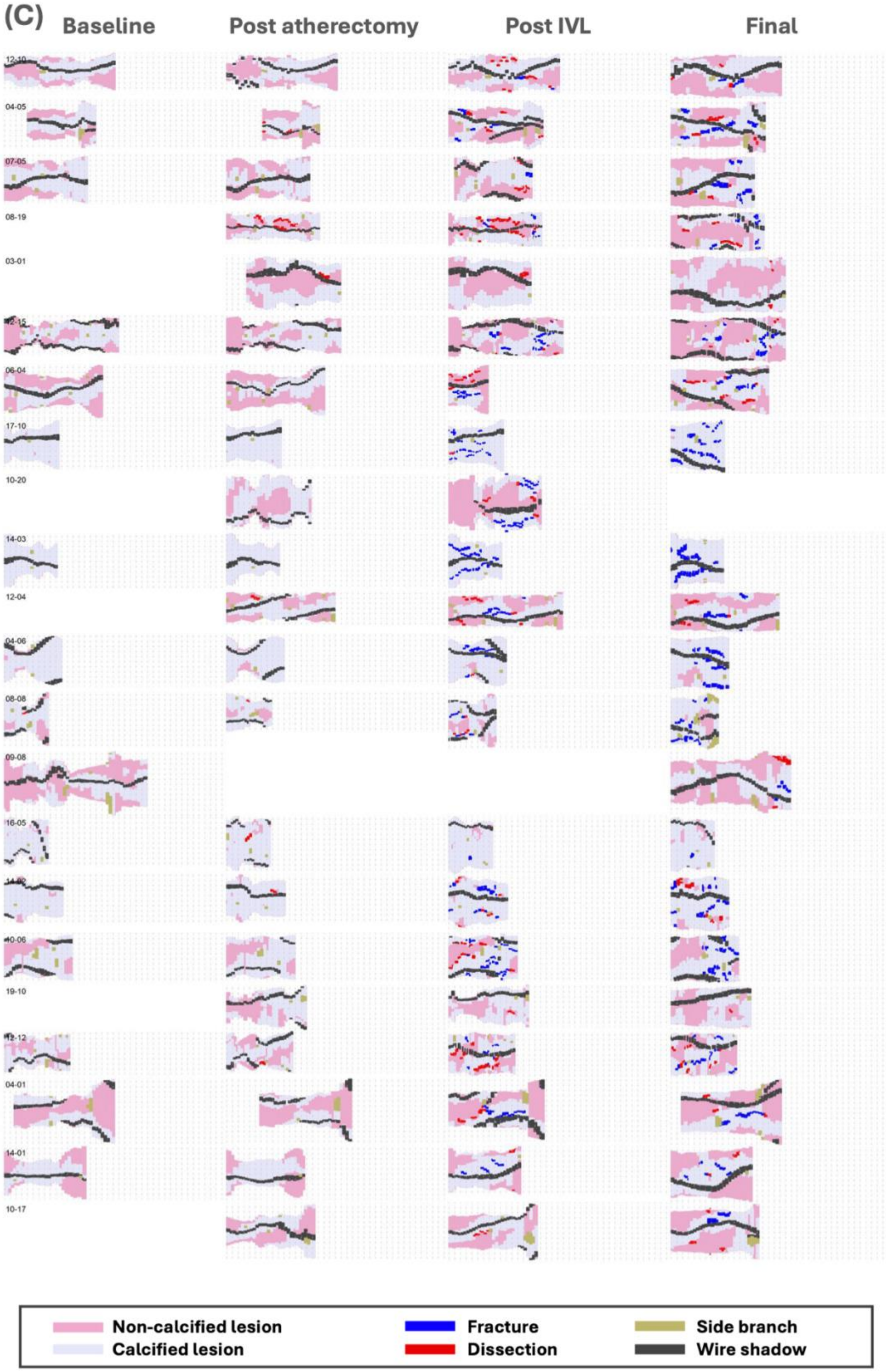
(C)
Baseline
Post atherectomy
Post IVL
Final
12-10
04-05
07-05
08-19
03-01
06-04
17-10
10-20
14-03
12-04
04-06
08-08
09-08
16-05
19-10
04-01
14-01
10-17
Non-calcified lesion
Calcified lesion
Fracture
Dissection
Side branch
Wire shadow

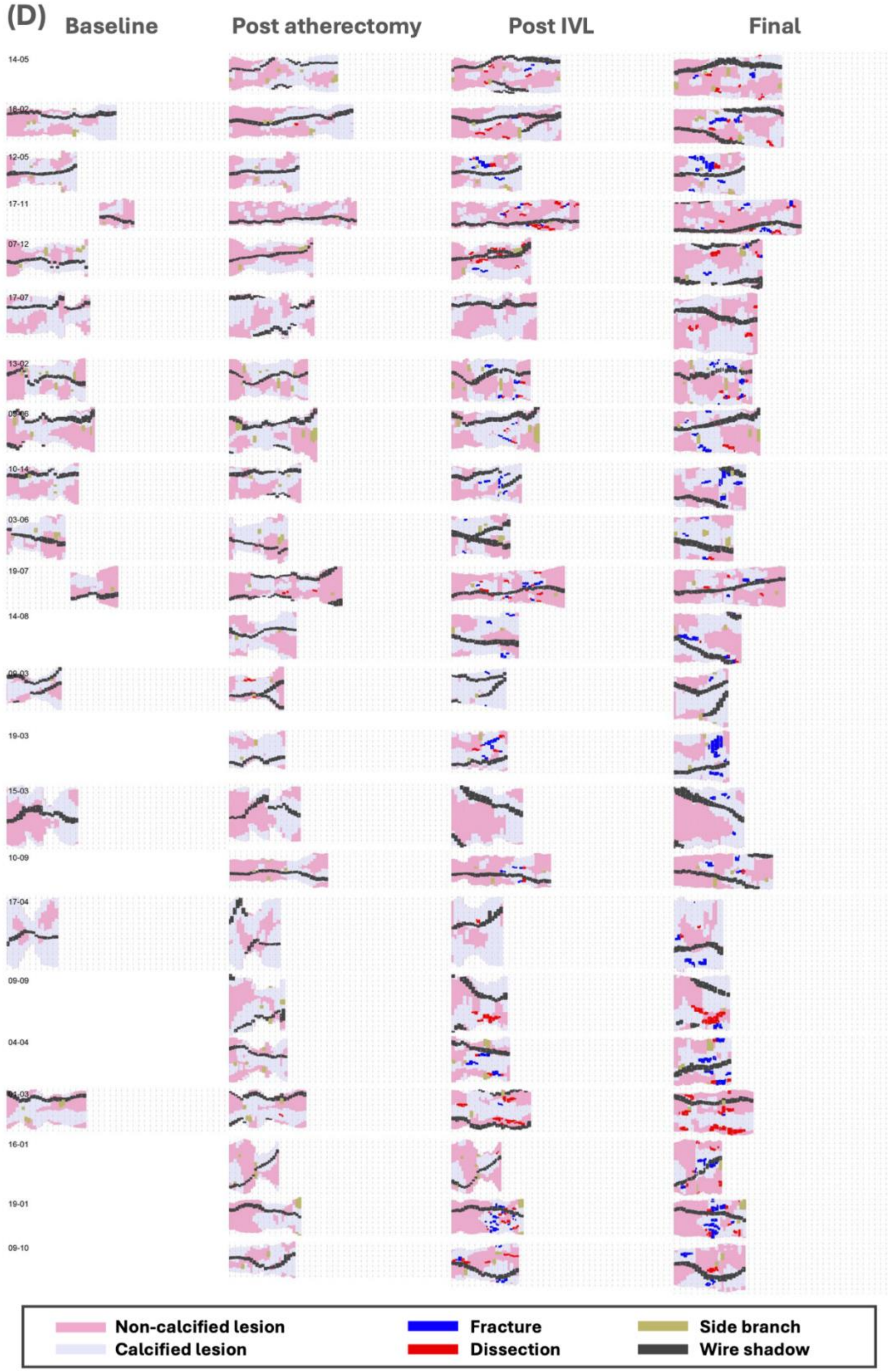
(D)
Baseline
Post atherectomy
Post IVL
Final
14-05
16-02
12-05
17-11
07-12
17-07
13-02
10-14
03-06
19-07
14-08
19-03
15-03
10-09
17-04
09-09
04-04
16-01
19-01
09-10
Non-calcified lesion
Calcified lesion
Fracture
Dissection
Side branch
Wire shadow

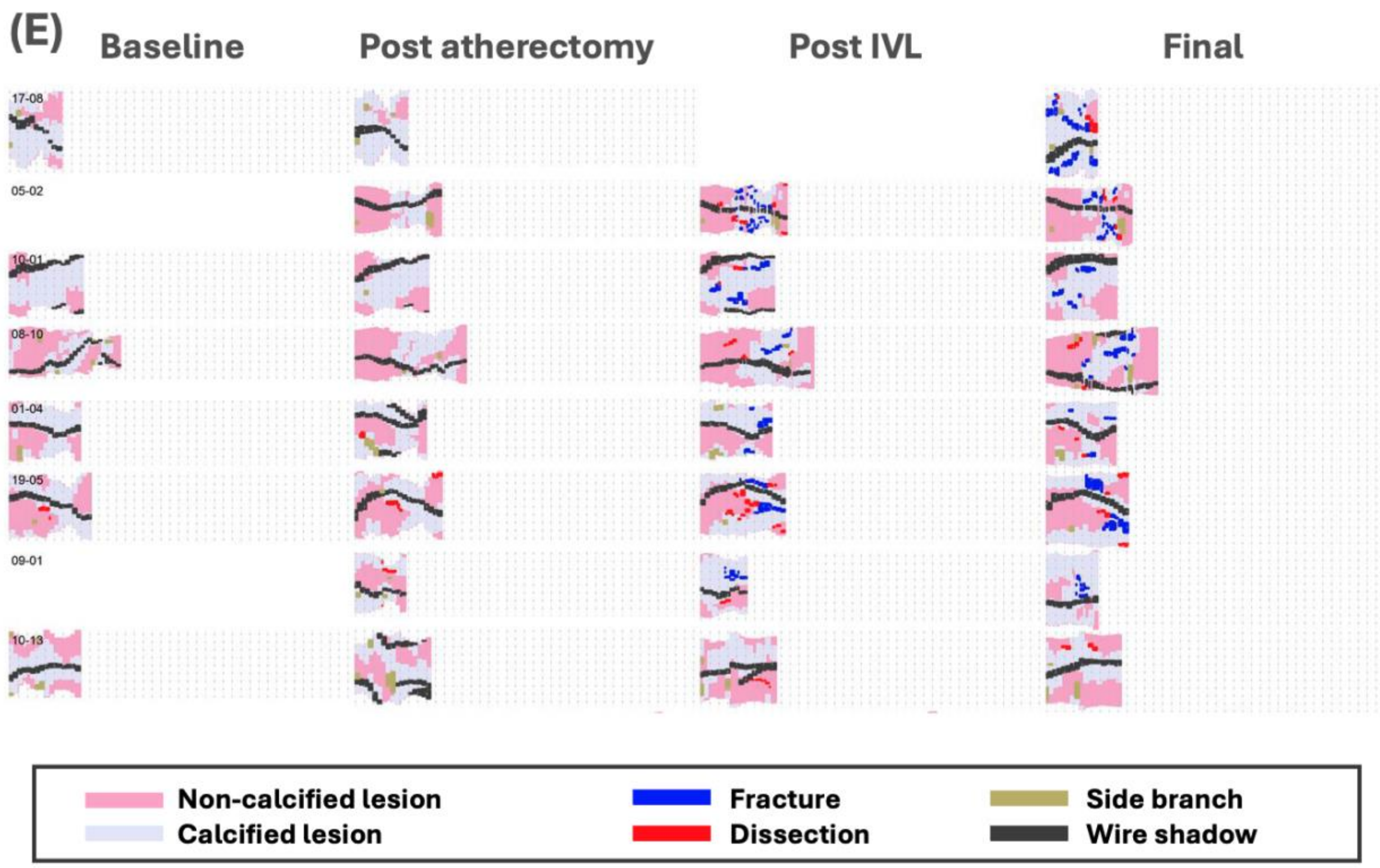
(E)
Baseline
Post atherectomy
Post IVL
Final
17-08
05-02
10-01
08-10
01-04
19-05
09-01
10-13
Non-calcified lesion
Calcified lesion
Fracture
Dissection
Side branch
Wire shadow

Figure S3. Spread-out vascular maps for eccentric spiral type calcification

The spread-out vascular map illustrates the circumferential and longitudinal distribution of calcified plaque, calcium fractures, and dissections throughout the dual preparation strategy (atherectomy followed by intravascular lithotripsy [IVL]) and subsequent stent deployment. Images are arranged chronologically from left to right: baseline, post-atherectomy, post-IVL, and Final. The horizontal axis represents the vessel's longitudinal direction (distal to proximal), while the vertical axis indicates circumferential plaque location reconstructed from OCT-derived angular data. The vertical extent at each longitudinal position corresponds to the lumen area, enabling visual assessment of luminal changes along the vessel.

Eccentric spiral-type lesions (n = 7) tend to fracture at the central body of the calcium, where balloon expansion can effectively deliver force.

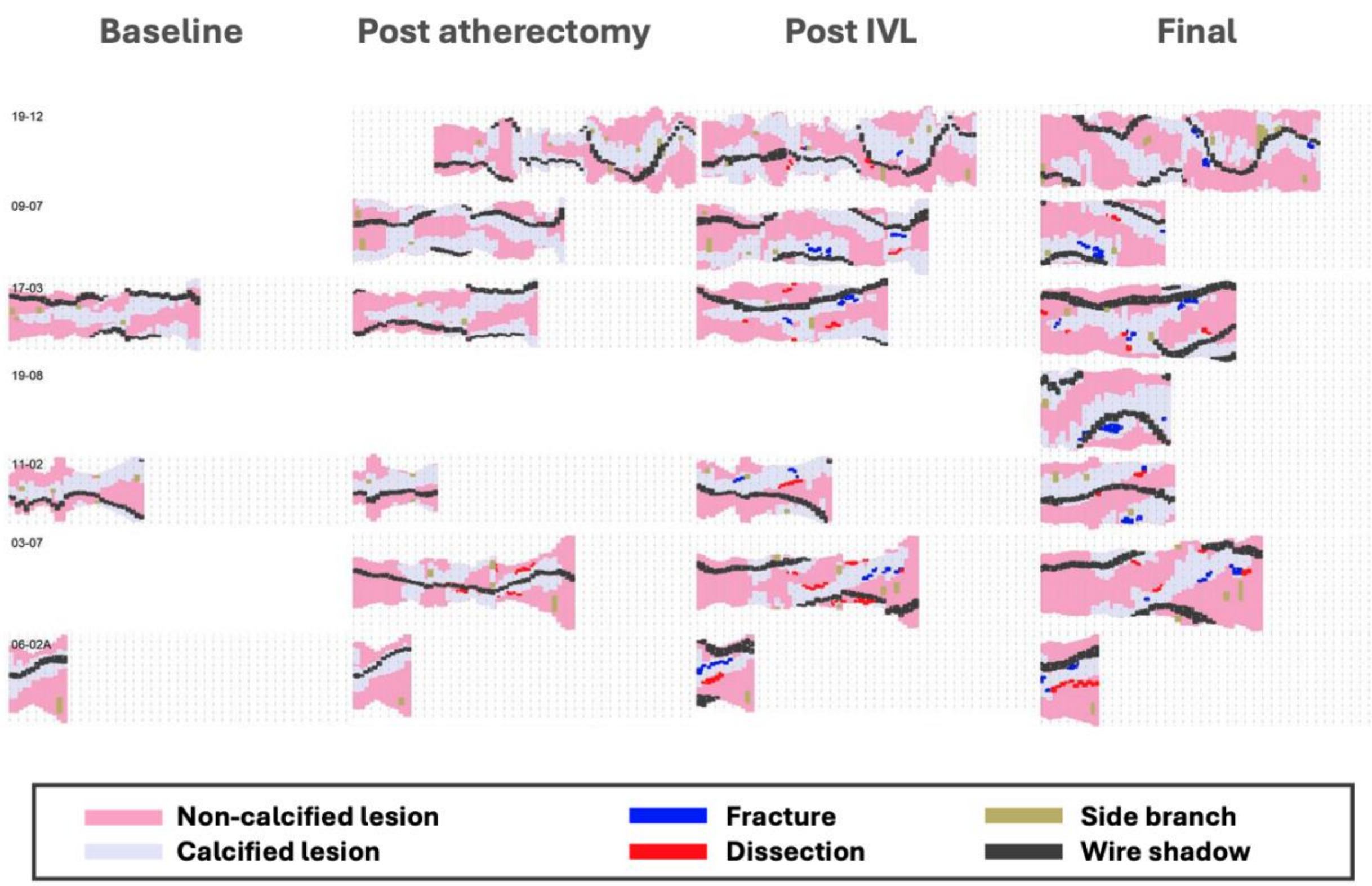

Figure S4. Spread-out vascular maps for eccentric straight type calcification

The spread-out vascular map illustrates the circumferential and longitudinal distribution of calcified plaque, calcium fractures, and dissections throughout the dual-preparation strategy (atherectomy followed by intravascular lithotripsy [IVL]) and subsequent stent deployment. Images are arranged chronologically from left to right: baseline, post-atherectomy, post-IVL, and Final. The horizontal axis represents the vessel's longitudinal direction (distal to proximal), while the vertical axis indicates circumferential plaque location reconstructed from OCT-derived angular data. The vertical extent at each longitudinal position corresponds to the lumen area, enabling visual assessment of luminal changes along the vessel.

Eccentric straight-type lesions (n = 7) are highly resistant to fracture, as the balloon force is not effectively transmitted to the calcified segment.

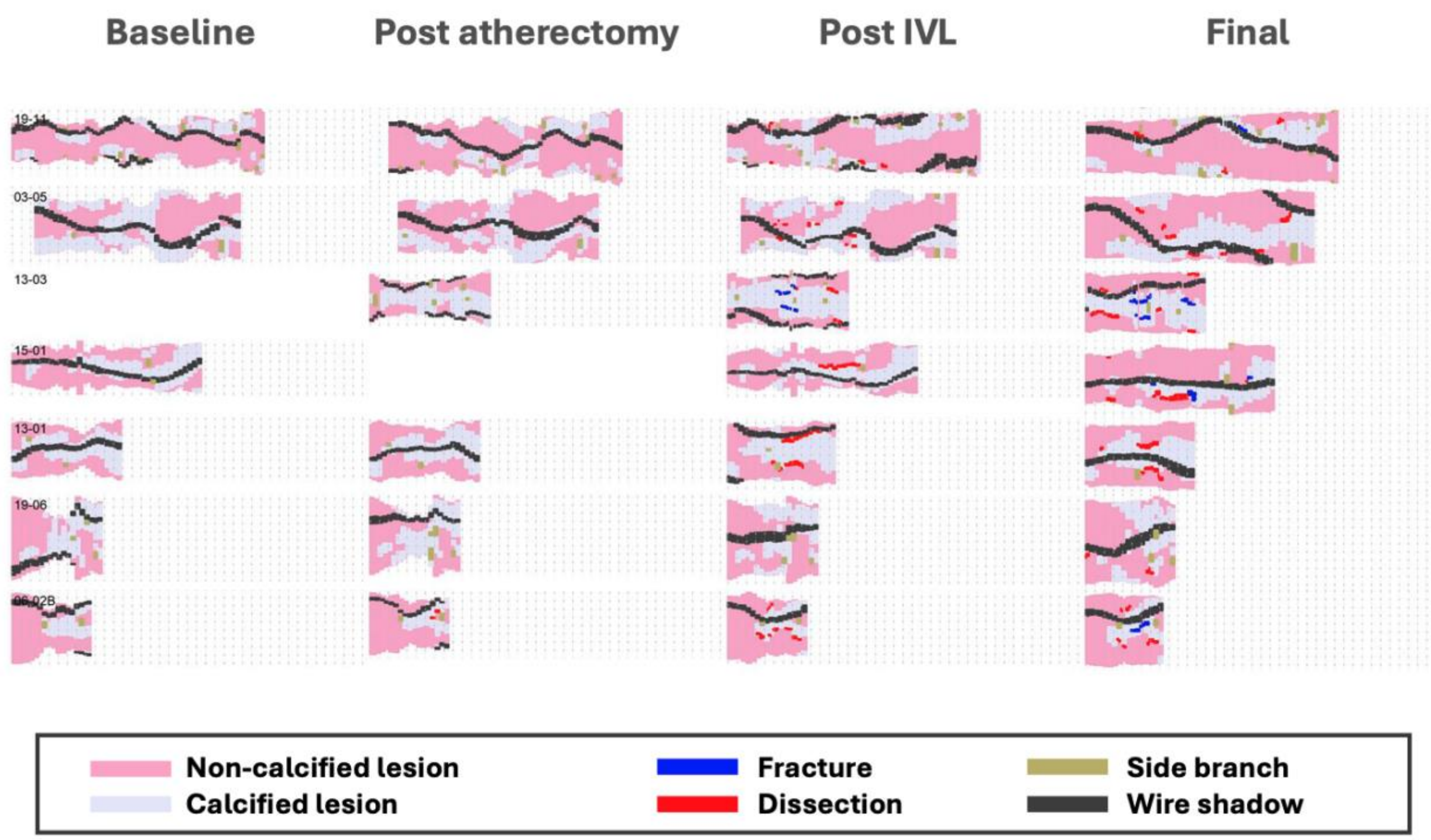

Supplemental Tables

Table S1. Patient demographics

	N = 114 patients
Age, years	75.87 (9.06)
Male	79 (69.3)
Body Mass Index	23.14 (3.75)
Diabetes mellitus	66 (57.9)
Hypertension	93 (81.6)
Dyslipidemia	87 (76.3)
Current smoker	7 (6.1)
Clinical presentation	
Stable angina	79 (69.3)
Silent myocardial ischemia	25 (21.9)
Unstable angina	4 (3.5)
ST-elevation myocardial infarction	1 (0.9)
Non-ST-elevation myocardial infarction	5 (4.4)
Prior myocardial infarction	20 (17.5)
History of stroke	22 (19.3)
History of percutaneous coronary intervention	46 (40.4)
History of coronary artery bypass graft	4 (3.5)
Atrial fibrillation	17 (14.9)
Ejection fraction, %	57.05 (10.80)
Estimated glomerular filtration rate, mL/min/1.73m^2	44.70 (27.14)

Data are expressed as mean (standard deviation) or number (percentage).

Table S2. Lesion characteristics

	N = 116 lesions
Target vessel	
Right coronary artery	36 (31.0)
Left anterior descending artery	75 (64.7)
Left circumflex artery	3 (2.6)
Left main trunk	6 (5.2)
Chronic total occlusion	2 (1.7)
Bifurcation lesion	30 (25.9)
Type B2/C lesion	116 (100)
Severe calcification on angiography[22]	116 (100)
Quantitative coronary angiography	
Lesion length, mm	34.36 (15.28)
Proximal reference diameter, mm	3.52 (0.56)
Distal reference diameter, mm	2.71 (0.66)
Minimum lumen diameter, mm	0.72 (0.28)
Percent diameter stenosis, %	72.40 (9.52)
Calcium length, mm	79.06 (20.75)
Optical coherence tomography	
Calcium score[4]	4.0 (0.0)
Calcified nodule[21]*	
None	50 (45.0)
Eruptive calcified nodule	29 (26.1)
Non-eruptive calcified nodule	32 (28.8)

Data are expressed as mean (standard deviation) or number (percentage).

*Calcified nodule data was available in 111 lesions.

Table S3. Procedural characteristics

	N = 114 patients
Approach site	
Radial approach	63 (55.3)
Distal radial approach	11 (9.6)
Femoral approach	36 (31.6)
Brachial approach	4 (3.5)
Size of guiding catheter	
6Fr guiding	35 (30.7)
7Fr guiding	77 (67.5)
8Fr guiding	2 (1.8)

Data are expressed as number (percentage). Other details are described in Figure 2.

Table S4. Firth's logistic regression analysis of factors associated with stent asymmetry and eccentricity

	Stent asymmetry (Asymmetry index ≥0.30)		Stent eccentricity (Eccentricity index ≤0.70)	
	N = 68/96 (70.8%)		N = 35/96 (36.5%)	
	OR [95%CI]	P value	OR [95%CI]	P value
Calcium length per 1 mm	1.05 [1.01, 1.10]	0.024	1.02 [0.98, 1.06]	0.315
Maximum calcium angle per 10 degrees	0.99 [0.83, 1.16]	0.857	1.04 [0.90, 1.21]	0.607
Mean calcium angle per 10 degrees	1.09 [0.98, 1.23]	0.129	0.98 [0.88, 1.07]	0.611
Maximum calcium thickness per 0.1 mm	0.95 [0.81, 1.11]	0.511	1.03 [0.90, 1.17]	0.675
IVL balloon artery ratio (IVL balloon/vessel diameter)	2.39 [0.04, 168.35]	0.683	0.58 [0.02, 19.61]	0.762
Stent artery ratio (Stent diameter/vessel diameter)	5.39 [0.12, 493.33]	0.406	2.74 [0.14, 61.78]	0.508
Number of calcium fracture after IVL	0.86 [0.58, 1.30]	0.467	0.93 [0.68, 1.27]	0.652
Rotational atherectomy†	1.13 [0.31, 3.94]	0.847	0.81 [0.26, 2.61]	0.725
Dual use (rotational atherectomy and orbital atherectomy) †	1.21 [0.04, 218.09]	0.917	0.99 [0.01, 29.52]	0.996
Non-eruptive calcified nodule‡	0.52 [0.17, 1.53]	0.232	1.39 [0.51, 3.77]	0.519
Eruptive calcified nodule‡	1.21 [0.34, 4.64]	0.775	1.90 [0.62, 5.99]	0.259
Calcium morphology: Eccentric spiral type§	13.01 [0.79, 2,547.56]	0.078	1.36 [0.19, 10.54]	0.754
Calcium morphology: Eccentric straight type§	0.85 [0.12, 5.75]	0.869	1.03 [0.14, 7.05]	0.975
Presence of medial dissection after IVL	1.27 [0.39, 4.02]	0.688	2.70 [0.88, 9.78]	0.085

Multivariable models were constructed using complete case analysis, whereby patients with missing values in any of the covariates were excluded from the analysis. *These variables are not included in the corresponding models. †With orbital atherectomy as the reference. ‡With lesions without calcified nodules as the reference. §With circumferential type as the reference. Abbreviations: OR, odds ratio; CI, confidence interval; IVL, intravascular lithotripsy.

Table S5. Unadjusted comparison of key OCT-derived outcomes according to atherectomy strategy

Variable	Rotational atherectomy group	Orbital atherectomy group	P value
Fracture after IVL	74/85 (87.1%)	11/17 (64.7%)	0.035
Fracture at Final	83/89 (93.3%)	17/19 (89.5%)	0.628
Calcium fracture per lesion after IVL	2.0 [1.0, 3.0]	1.0 [0.0, 2.0]	0.015
Calcium fracture per lesion at Final	3.0 [2.0, 4.0]	2.0 [2.0, 2.5]	0.045
Minimum stent expansion index	0.81 [0.70, 0.93]	0.89 [0.77, 0.94]	0.127
Stent underexpansion (<0.80)	40/89 (44.9%)	7/19 (36.8%)	0.614
Asymmetry index	0.37 [0.30, 0.47]	0.36 [0.28, 0.42]	0.357
Stent asymmetry (Asymmetry index ≥0.30)	67/89 (75.3%)	13/19 (68.4%)	0.569
Minimum eccentricity index	0.72 [0.66, 0.79]	0.72 [0.67, 0.76]	0.440
Stent eccentricity (Eccentricity index ≤0.70)	33/89 (37.1%)	8/19 (42.1%)	0.796

Data are expressed as number (percentage) or median [interquartile range]. Comparisons are unadjusted and intended to complement multivariable analyses. One lesion treated with both rotational and orbital atherectomy were excluded from this direct two-group comparison. Abbreviations: IVL, intravascular lithotripsy.

Table S6. Calcium fracture characteristics on Final OCT by calcium morphology subtype

	Circumferential type	Eccentric spiral type	Eccentric straight type	P value
Number of calcium fracture per lesion, n	3.0 [2.0, 4.0]	2.0 [1.5, 2.5]	1.0 [0.0, 1.5]	0.002
Minimum fracture depth, mm	0.18 (0.11)	0.16 (0.10)	0.20 (0.09)	0.811
Maximum fracture depth, mm	0.86 (0.46)	0.55 (0.28)	0.40 (0.24)	0.038
Mean fracture depth, mm	0.45 (0.21)	0.36 (0.16)	0.28 (0.11)	0.151
Minimum fracture length, mm	1.19 (1.10)	1.08 (0.90)	1.41 (0.95)	0.886
Maximum fracture length, mm	5.03 (2.96)	4.69 (3.14)	2.76 (2.30)	0.324
Mean fracture length, mm	2.69 (1.28)	2.83 (1.92)	1.88 (0.88)	0.460

Data are expressed as mean (standard deviation) or median [interquartile range]. P values were calculated using one-way ANOVA for variables presented as mean (SD) and the Kruskal–Wallis test for variables presented as median [interquartile range].
